# Effector Binding Sequentially Alters KRAS Dimerization on the Membrane: New Insights Into RAS‐Mediated RAF Activation

**DOI:** 10.1002/advs.202401530

**Published:** 2024-08-13

**Authors:** Soo‐Yeon Lee, Hyun‐Jong Eun, Ki‐Young Lee

**Affiliations:** ^1^ Department of Pharmacy College of Pharmacy and Institute of Pharmaceutical Sciences CHA University Pocheon‐si Gyeonggi‐Do 11160 Republic of Korea; ^2^ Research Institute of Pharmaceutical Sciences College of Pharmacy Seoul National University Seoul 08826 Republic of Korea; ^3^ School of Pharmacy Sungkyunkwan University Suwon 16419 Republic of Korea

**Keywords:** KRAS dimerization, nanodisc, paramagnetic relaxation enhancement, RBD‐CRD, sequential binding

## Abstract

RAS proteins are peripheral membrane GTPases that activate multiple downstream effectors for cell proliferation and differentiation. The formation of a signaling RAS–RAF complex at the plasma membrane is implicated in a quarter of all human cancers; however, the underlying mechanism remains unclear. In this work, nanodisc platforms and paramagnetic relaxation enhancement (PRE) analyses to determine the structure of a hetero‐tetrameric complex comprising KRAS and the RAS‐binding domain (RBD) and cysteine‐rich domain (CRD) of activated RAF1 are employed. The binding of the RBD or RBD–CRD differentially alters the dimerization modes of KRAS on both anionic and neutral membranes, validated by interface‐specific mutagenesis. Notably, the RBD binding allosterically generated two distinct KRAS dimer interfaces in equilibrium, favored by KRAS free and in complex with the RBD–CRD, respectively. Additional interactions of the CRD with both KRAS protomers are mutually cooperative to stabilize a new dimer configuration of KRAS bound to the RBD–CRD. The RAF binding sequentially alters KRAS dimerization, providing new insights into RAF activation, including a configurational transition of the KRAS dimer to provide an interaction site for the CRD and release the autoinhibited RAF complex. These methods are applicable to many other signaling protein complexes on the membrane.

## Graphical abstract

Nanodisc platforms and paramagnetic relaxation enhancement analyses revealed that the binding of the RAS‐binding domain (RBD) generates two distinct KRAS dimer interfaces in equilibrium, favored by KRAS free and in complex with the RBD–cysteine‐rich domain (CRD). Additional interactions of the CRD with both KRAS protomers are mutually cooperative to stabilize a new dimer conformation of KRAS.

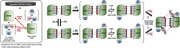

## 1 Introduction

RAS proteins (HRAS, KRAS and NRAS) are membrane‐associated small GTPases and act as molecular switches that cycle between the active GTP‐bound and inactive GDP‐bound states, regulating multiple intracellular signaling cascades for cell proliferation, differentiation and survival.^[^ ^1^ ^]^ The guanine nucleotide exchange factor (GEF) proteins activate RAS by catalysing the release of GDP and binding of GTP, while GTP hydrolysis is promoted by the GTPase activating proteins (GAPs). Notably, RAS mutations near the nucleotide binding site impair the GTPase activity and lock the protein in the active state, occurring in a quarter of human cancers. Among RAS isoforms, KRAS is mutated in ∼80% of RAS‐driven cancers.^[^ ^2^ ^]^ Activated RAS proteins prefer to interact with downstream RAF effectors and initiate mitogen‐activated protein kinase (MAPK) pathway for oncogenesis.^[^ ^3^ ^]^ In addition, transient dimers or higher‐order oligomers of KRAS at the plasma membrane have been proposed to act as scaffolds to promote the proximity‐driven dimerization and activation of RAFs.^[^ ^4^ ^]^ Raf consists of the N‐terminal Ras binding domain (RBD) and the cysteine‐rich domain (CRD), which is responsible for both membrane association and RAS binding, the C‐terminal kinase domain (KD) and a long linker (∼160 amino acids) between KD and CRD. Recent studies have revealed structural snapshots of monomeric RAFs in the autoinhibited state, active RAF dimers, the hetero‐dimeric complex between RAS and the RBD–CRD, and effector‐free RAS dimers on the membrane,^[^ ^5^ ^]^ representing great progress in mechanistic understanding of RAS‐mediated RAF activation at the plasma membrane. A feasible mechanistic model is that autoinhibited RAFs in the cytosol are recruited to the plasma membrane by binding to RAS nanoclusters, likely comprising dimers or higher‐order oligomers, at the plasma membrane. This RAS binding may promote an open, active conformation of RAF and enable the KDs to dimerize and exert their kinase activity. Alternatively, it is possible that the hetero‐tetrameric RAS‐RAF complex is formed through dimerization of preformed RAS–RAF heterodimers, supported by a MD simulation showing that RBD binding allosterically promotes RAS dimerization.^[^ ^6^ ^]^ However, despite recently reported several structures of RAS and RAF, how bindings of the RBD or RBD–CRD domains impact plastic modes KRAS dimerization on the membrane and the formation of a signaling RAS:RAF complex remains unknown.

Herein, we employed nanodisc‐based systems to determine the structure of KRAS‐GTPγS (a non‐hydrolysable GTP analogue) dimers in complex with the RAF1 RBD–CRD on the membrane using paramagnetic relaxation enhancement (PRE)‐NMR. PRE experiments have been developed as highly sensitive tools for studying low‐affinity protein complexes at the atomic resolution.^[^ ^7^ ^]^ Nanodiscs provide a stable native lipid‐bilayer membrane of defined size and composition, encapsulated by two copies of membrane scaffold protein (MSP).^[^ ^8^ ^]^ Selective isotope labeling methods were employed to effectively overcome challenges with line broadening and overlap of NMR cross‐peaks from the KRAS: RBD–CRD complex anchored to nanodiscs with a molecular weight of >150 kDa. We found that bindings of either RBD or RBD–CRD domains promotes KRAS dimerization on membranes containing or lacking anionic phosphatidylserine (PS) lipids, along with altered interprotomer orientations at the “α‐α” dimer interface comprising two α4‐α5 surfaces of KRAS. Newly formed interactions at the dimer interface are observed at a crystal contact between RAS GTPase domains incapable of membrane association. Interface‐specific mutagenesis demonstrated that binding of the RBD alone induces conformational equilibrium between two KRAS dimers resembling those free and in complex with the RBD–CRD, respectively. Additional bindings of the CRD to both KRAS protomers are mutually cooperative to stabilize the hetero‐tetrameric KRAS: RBD–CRD complex on the membrane. The effector‐dependent modulation of KRAS dimerization, presented here, advances the structural model for the assembly process of a signaling KRAS‐RAF complex.

## 2 Results and Discussion

### 2.1 Effector Binding Alters the Dimerization Interface of KRAS on the Membrane

Previous PRE experiments have revealed plastic, reversible “α‐α” dimerization interfaces of KRAS on the membrane, involving alternate electrostatic interactions between R135 and either D153 or E168 at the dimer interface.^[^ ^5f^ ^]^ The dimerization is modulated by the nucleotide and mutation states of KRAS and the lipid composition of the membrane.^[^ ^5^, ^9^ ^]^ However, it remains unknown how this plastic mode of dimerization is altered by binding of the RBD or the tandem RBD–CRD domain of activated RAF kinase, which is a key downstream effector of RAS.

To probe the impact of RAF binding on the KRAS dimer interface, we measured intermolecular PRE effects between activated, fully processed KRAS molecules (i.e., the C‐terminally farnesylated and carboxy‐methylated form, henceforth referred to as KRAS). KRAS or the heterodimeric complex between KRAS and the RBD or RBD–CRD of RAF1 were selectively isotopically ^13^C‐labeled at single methyl groups in Ile, Leu and Val (ILV, Cδ1, Cδ, and Cγ, respectively) and mixed at a 1:1 molar ratio with the same construct that was tagged with TEMPO [(2,2,6,6‐Tetramethylpiperidin‐1‐yl)oxyl] spin labels at Cys118 or Cys169 at the periphery of the α‐interface of KRAS, in the presence of MSP1E3D1‐type nanodiscs either with or without 20% PS. Two molar ratios of KRAS, KRAS–RBD, or KRAS: RBD–CRD to the nanodisc leaflet (100 µM) were used to promote their dimerization on the membrane. PS is the most abundant anionic phospholipid in eukaryotic membranes and promotes the formation of KRAS dimers or higher‐order oligomers to recruit and activate downstream RAF effectors at the plasma membrane.^[^ ^10^ ^]^ However, although nanodiscs provide fragments of native‐like planar membranes of defined lipid compositions, reconstituting dynamically altered lipid compositions of the plasma membrane is challenging.

**Figure 1:**
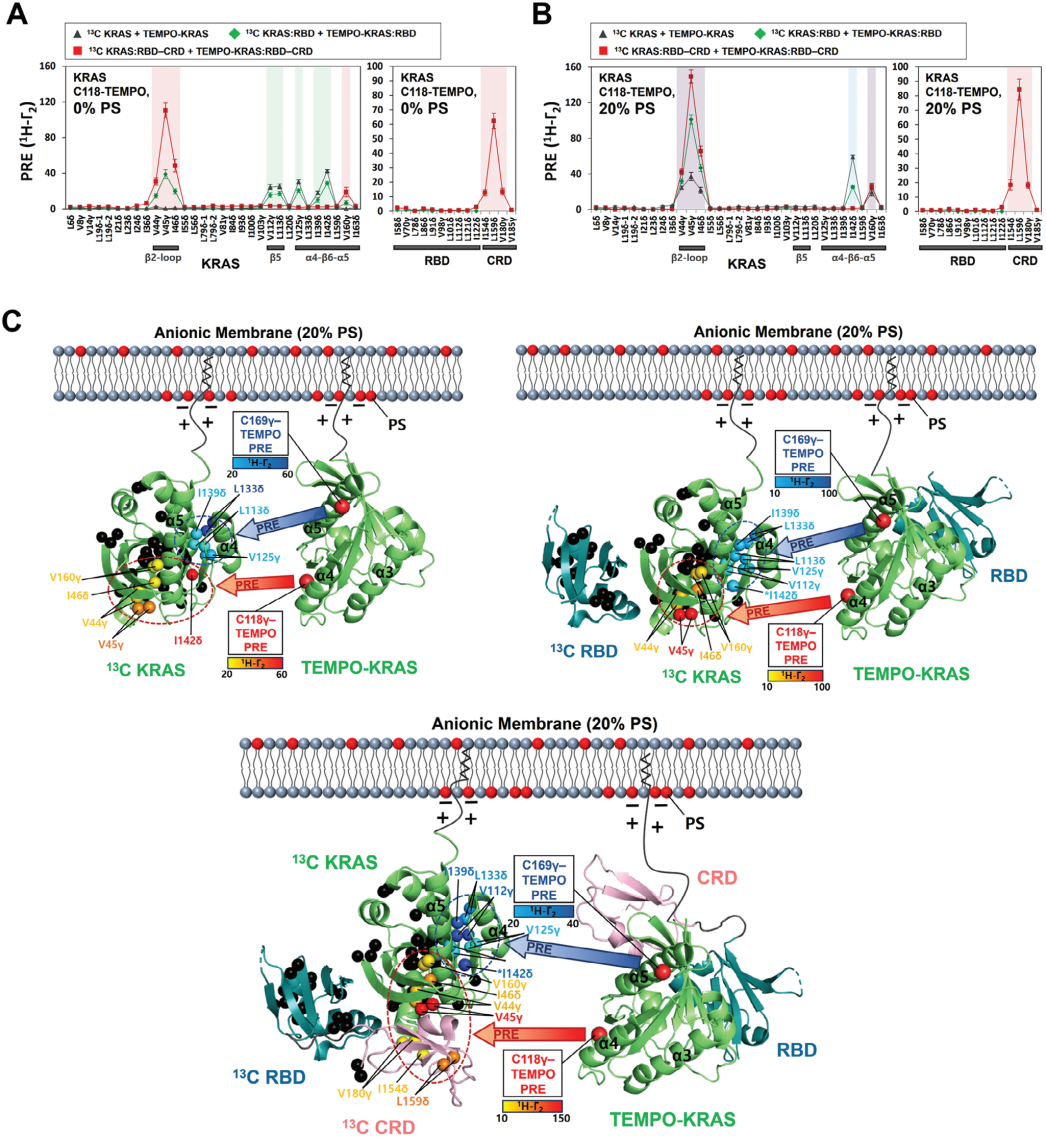
Effector‐dependent modulation of KRAS dimerization on membranes lacking or containing 20% PS. PRE rates for ILV ^13^C‐methyl probes in KRAS, KRAS: RBD, and KRAS: RBD–CRD induced by the addition of the same construct bearing a TEMPO spin label at Cys118 in KRAS in the presence of nanodiscs either containing A) or lacking B) PS. Plots obtained for KRAS, KRAS: RBD, and KRAS: RBD–CRD are colored black, green, and red, respectively. The PRE effects for effector‐free KRAS dimers on membranes lacking or containing 20% PS and those for the KRAS: RBD–CRD complex on both membranes are indicated by rectangular boxes shaded in green, blue, and red, respectively. Overlay of the PRE effects for free and effector‐bound KRAS dimers is shown in the box shaded in violet. C) Mapping dimerization‐induced PRE effects onto the structures of KRAS, KRAS: RBD, and KRAS: RBD–CRD on anionic membranes containing 20% PS. Probes that exhibited measurable PREs are colored according to the magnitude of the PRE rate (^1^H‐Γ_2_) as indicated, and PRE‐unaffected probes are colored in dark gray. Arrows represent PRE effects that arose from TEMPO conjugated to Sγ atoms of Cys118 (red) and Cys169 (blue) in the opposing KRAS protomer.

Intermolecular PRE effects were induced by dimerization of KRAS free or in complex with the RBD or RBD–CRD of RAF1 (i.e., preformed KRAS:RBD or KRAS: RBD–CRD heterodimers) on the surface of lipid nanodiscs. In the absence of TEMPO spin labels, no obvious chemical shift perturbations nor obvious differences in intensity reductions of peaks were observed upon the addition of nanodiscs (Figures S1 and S2, Supporting Information), likely because strong NMR signals from freely diffusing KRAS: RBD–CRD in solution obscured signals from membrane‐associated KRAS: RBD–CRD. Representative spectra with peaks that exhibit differential PRE effects are shown in Figures S3–S7 (Supporting Information). We measured ^1^H transverse PRE rates (^1^H‐Γ_2_) for the ILV ^13^C‐methyl probes, and these values were plotted by residue (**Figure** **1**; Figure S8, Supporting Information). The patterns of PRE effects for the α‐interface of effector‐free KRAS on both anionic and neutral membranes are substantially distinct from those obtained with KRAS in complex with either the RBD or RBD–CRD. These data demonstrated that binding of these RAF subdomains allosterically alters the “α‐α” dimer interface on the opposite side of the β‐sheet effector binding site (β‐interface) of KRAS, supported by recent molecular dynamics (MD) simulations in which the KRAS: RBD interaction promotes KRAS dimerization by allosteric communication between two terminal RBDs of the KRAS‐RBD hetero‐tetramer.^[^ ^6^ ^]^ Notably, the PRE data for the RBD‐bound KRAS dimers appear to comprise a “mixed” PRE profile, intermediate between those obtained with and without the RBD–CRD, regardless of the presence of PS in the membrane. Additionally, ^13^C‐labeled KRAS: RBD–CRD exhibited measurable PRE effects for several probes (I154δ, L159δ, and V180γ) in the CRD domain upon addition of KRAS: RBD–CRD in which KRAS is tagged with a TEMPO spin label at Cys118, but not Cys169. However, the interaction between TEMPO‐labeled KRAS and ^13^C‐labeled RBD–CRD at the β‐interface did not cause any PRE effects for the RBD–CRD (Figure S9, Supporting Information). These observations demonstrate that the CRD is close or bound to the α‐interface of the opposing KRAS protomer within the hetero‐tetrameric KRAS: RBD–CRD complex on the membrane. Notably, unlike KRAS and KRAS: RBD constructs, KRAS: RBD–CRD on both anionic and neutral membranes exhibited high similarity in overall patterns of PRE effects for the dimer interface, with higher PRE effects obtained with the anionic membrane (Figure 1; Figure S8, Supporting Information). These data demonstrated that the PS effect on KRAS dimerization is abolished by binding of the RBD–CRD to KRAS protomers and that the RBD–CRD‐dependent modulation of KRAS dimerization is more pronounced in the presence of PS. However, no intermolecular PRE effects between KRAS molecules in complex with either the RBD or the RBD–CRD were observed in the absence of nanodiscs (Figure S10, Supporting Information), demonstrating that dimerization of the KRAS–effector heterodimer is membrane‐dependent. Mass spectrometry was used to confirm that KRAS purified from insect cells was farnesylated and methylated at the C‐terminal membrane anchor (Figure S11, Supporting Information).

To examine the effect of NaCl on dimerization of the KRAS: RBD–CRD complex on the membranes containing or lacking PS, we performed TEMPO‐PRE experiments in the presence of 150 mm NaCl and compared the PRE effects obtained with 150 and 300 mm NaCl. The lower NaCl concentration induced overall increases in the TEMPO‐PRE effects for ^13^C‐labeled KRAS: RBD–CRD, and the effect of NaCl was prominent in the presence of PS (Figure S12, Supporting Information), while preserving the PRE patterns. This is likely due to an increase in electrostatic interactions for the membrane association and subsequent dimerization of KRAS: RBD–CRD. Although the PRE methodology used in this study can detect transient dimers of KRAS in its free form or complexed with RBD or RBD–CRD very sensitively, it predominantly detects the major species and the magnitude of PRE depends on the placement of spin labels on Cys residues. Thus, the possibility of other dimer interfaces with much shorter lifetimes should not be excluded.

### 2.2 Mutagenesis‐Based Selection of a New Dimerization Interface of KRAS

To probe whether the observed PRE for the dimer interface of KRAS in complex with the RBD or the RBD–CRD is simply a mixture of PREs arising from several dimer interfaces or are derived from a new single‐dimer interface, we first employed interface‐specific E168R and D153K mutants of KRAS, which were shown to selectively disrupt nanodisc‐tethered KRAS dimers on anionic and neutral membranes, respectively.^[^ ^5f^ ^]^ Each mutation was introduced into both ^13^C‐ and TEMPO‐labeled KRAS free or in complex with the RBD or RBD–CRD, and the aforementioned experiments were performed to observe the intermolecular PRE between the same KRAS mutants. Both E168R and D153K mutants of effector‐free KRAS selectively abolished the PRE effect for the corresponding α‐α dimer interface (Figure S13, Supporting Information). Interestingly, the introduction of E168R or D153K into the KRAS: RBD complex on anionic and neutral membranes, respectively, induced equilibrium shifts toward the overall PRE pattern obtained with the KRAS: RBD–CRD complex. These observations demonstrate that the KRAS dimer interfaces resembling those of effector‐free and RBD–CRD‐bound KRAS co‐exist in the RBD‐bound state of KRAS, regardless of the PS composition in the membrane, and that this interfacial equilibrium can be readily spoiled by single‐point mutations. By contrast, the PRE profiles for KRAS: RBD–CRD on both membranes were not affected by both E168R and D153K mutations in KRAS (Figure S13, Supporting Information), demonstrating that the additional binding of the CRD fully shifts the equilibrium toward a new KRAS dimer, distinct from effector‐free KRAS dimers that are sensitive to these mutations.

**Figure 2:**
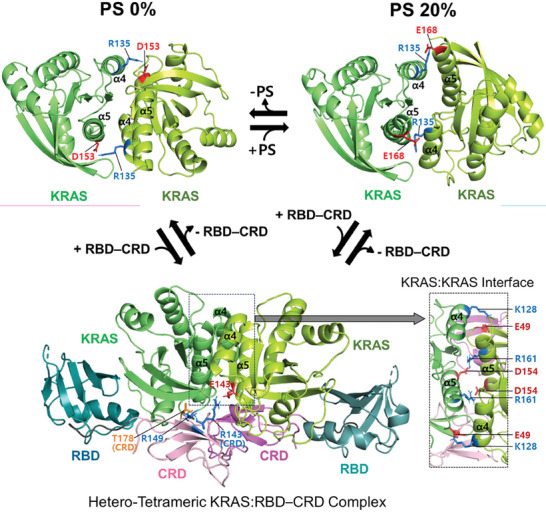
Representative PRE‐derived structures of membrane‐bound KRAS dimers unbound versus bound to the tandem RBD–CRD domain of RAF1. The dimerization interface of effector‐free KRAS is modulated by the PS lipid composition in the membrane and involves alternative electrostatic interactions of R135 with D153 and E168 at the α4‐α5 dimer interface. The binding of the RBD–CRD to KRAS protomers induces a new dimer interface involving the E49–K128 interaction at the KRAS: KRAS interface and the E143–R143 interaction at the KRAS: CRD interface, regardless of the lipid composition. These electrostatic interactions and the interaction between R149 in KRAS and T178 in CRD at the KRAS: RBD–CRD interface were validated by mutagenesis in this study.

Based on the sets of PRE‐derived intermolecular distances between two KRAS protomers and between a KRAS protomer and the opposing CRD within the dimer interface of the KRAS: RBD–CRD complex, we constructed the structural model of the hetero‐tetrameric KRAS: RBD–CRD complex in which two KRAS protomers bind to the RBD–CRD of RAF1 (**Figure** **2**; Table S1, Supporting Information). Different sets of distances were calculated from the faction of the dimer (F_dimer_), and the NOE distance restraint potential (E_noe_) values for the 200 lowest HADDOCK‐score structures were plotted versus the F_dimer_ values, indicating that KRAS: RBD–CRD dimer model with the 0.25 value is optimal (Figure S14, Supporting Information). The 20 lowest‐energy structures are well converged in a single cluster with an average RMSD value of 0.94 ± 0.18 Å (Figure S15, Supporting Information). There are no violations of PRE‐derived distances in the 20 structures with a low Q‐ factor of 0.1 (Table S2, Supporting Information), suggesting that the observed PRE profile for KRAS: RBD–CRD reflects a single dimerization interface of KRAS in complex with the RBD–CRD. The structural models generated from the best‐fitting distances (F_dimer_ = 0.25) and the worst‐fitting distances (F_dimer_ = 1.0) are converged with an RMSD value of 1.13, indicating that they are essentially the same (Figure S16, Supporting Information). Notably, these structures are distinct from those as we previously determined in the absence of the RBD–CRD^[^ ^5f^ ^]^ (Figure 2), in the relative orientations of KRAS protomers at the “α‐α” interface. PRE‐driven structures of the KRAS dimer in complex with the RBD–CRD of RAF1 are stabilized mainly by intermolecular electrostatic interactions between i) K128 and E49 and ii) D154 and R161 at the KRAS‐KRAS interface and between E143 of KRAS and R143 of the opposing CRD, within the dimer interface of the KRAS: RBD–CRD complex (Figure 2; Table S3, Supporting Information). Remarkably, the KRAS dimer interface is almost identical, with an average RMSD of 1.24 ± 0.21 Å, to previously reported crystallographic contacts of the truncated form of KRAS, although this construct lacks the disordered C‐terminal membrane anchor and is crystallized in the absence of the membrane (Figure S17, Supporting Information). Previously reported MD simulation data have shown that diverse KRAS oligomers can be formed using multiple weak‐affinity interfaces involving two partially overlapping α3/α4 and α4/α5 regions.^[^ ^11^ ^]^ However, the three α–α dimerization interfaces of KRAS in its free form and complexed with the RBD–CRD of RAF1 on the membrane (Figure 2), as determined from our PRE experimental data, largely overlap. Thus, higher‐order self‐assembly involving combinations of these interfaces might not occur.

**Figure 3:**
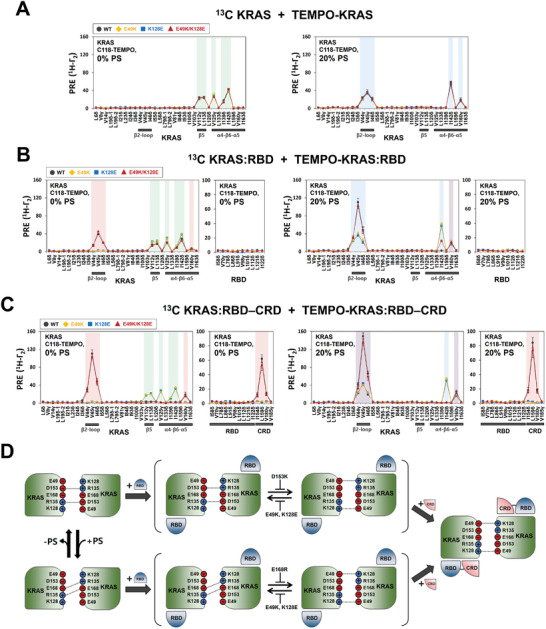
Mutagenic validation of the intermolecular interaction specific to the dimer interface of KRAS in complex with the tandem RBD–CRD domain of RAF1 on the membrane. PRE rates for ILV ^13^C‐methyl probes in KRAS A), KRAS: RBD B) or KRAS: RBD–CRD C) and their interfacial charge‐reversal mutants (E49K, K128E and E49K/K128E in KRAS) were measured in the presence of the same construct with a TEMPO spin label at Cys118 and the membrane lacking or containing 20% PS, as indicated. Plots obtained with wild‐type KRAS and the E49K, K128E, and E49K/K128E mutants are colored black, yellow, blue, and red, respectively. The PRE effects for effector‐free KRAS dimers on membranes lacking or containing 20% PS and those of the KRAS: RBD–CRD complex on both membranes are indicated by rectangular boxes shaded in green, blue, and red, respectively. Overlay of the PRE effects for free and effector‐bound KRAS dimers is shown in the box shaded in violet. D) Schematic of the effector‐dependent modulation of intermolecular electrostatic interactions at the KRAS dimer interface in the presence or absence of PS and effects of the interfacial single and double charge‐reversal mutations on the equilibrium between two dimer states of KRAS in complex with the RBD. This equilibrium involves alteration between R135–D153 (top) or R135–E168 (bottom) interactions and the E49–K128 interaction at the KRAS dimer interface, and the latter interaction is promoted by additional binding of the CRD to KRAS protomers.

To validate the structural model of the KRAS dimer bound to the RBD–CRD, we introduced charge‐reversal mutations at key points of interaction specific to the dimerization interface of KRAS in complex with the RBD–CRD. Consistent with the model, experiments with both E49K and K128E mutants of KRAS selectively eliminated the corresponding PRE effect in profiles induced by dimerization of KRAS: RBD or KRAS: RBD–CRD, in the presence of both anionic and neutral membranes, whereas the PRE effects for effector‐free KRAS dimers were not affected by these mutations (**Figure** **3**; Figure S18, Supporting Information). These results demonstrate that residues E49 and K128 are specifically involved in KRAS dimerization in the effector‐bound states. In both KRAS: RBD and KRAS: RBD–CRD profiles, these mutants exhibited an increase in the overall PRE pattern obtained with effector‐free KRAS, indicative of an equilibrium shift from the effector‐bound to free KRAS dimer. Furthermore, the reduced PRE effects were rescued by double charge‐reversal E49K/K128E mutants, likely due to the recovery of the charge complementarity between these residues at the KRAS: KRAS interface (Figure 3; Figure S18, Supporting Information), highlighting the importance of the E49‐K128 electrostatic interaction in dimerization of the KRAS: RBD or KRAS: RBD–CRD complexes. These mutagenesis data are fully consistent with differences between dimerization modes of KRAS free and in complex with the RBD and RBD–CRD on the membrane. The effector‐dependent modulation of KRAS dimerization provides new structural insights into the RAF activation process involving the interaction between membrane‐associated KRAS and the RAS binding tandem domain (RBD–CRD) of RAF.

### 2.3 The CRD Dynamics Impacts Dimerization of the KRAS: RBD–CRD Complex on the Membrane

The CRD of RAFs has been recognized as a critical regulatory element to interact with the RAF KD and the 14‐3‐3 protein in the closed, autoinhibited complex of RAF[5a‐c], as well as KRAS and anionic membranes during RAF activation.^[^ ^5^, ^12^ ^]^ However, in contrast to the well‐established importance of the RBD in the high‐affinity interaction between RAS and RAF,^[^ ^3b^ ^]^ it remains unclear how much the CRD domain contributes to the RAS‐RAF binding and the formation of a signaling RAS: RAF complex on the membrane.^[^ ^5g^ ^]^ Recent MD simulations have shown that the CRD is highly dynamic in the context of the KRAS: RBD–CRD complex and that this dynamic is reduced when the complex is associated with the membrane.^[^ ^13^ ^]^ The KRAS: CRD interaction depends on the high‐affinity anchoring of the RBD to KRAS.^[^ ^12^ ^]^ However, it remains unknown how the dynamic KRAS: CRD binding impacts plastic modes of KRAS dimerization, and vice‐versa.

To probe how dimerization of the KRAS: RBD–CRD complex influences the preformed interaction between KRAS and the RBD–CRD, we measured the intermolecular PRE between ^13^C‐labeled RBD–CRD and KRAS labeled with TEMPO at Cys1 near the β‐interface upon addition of isotopically unlabeled KRAS: RBD–CRD to favor dimerization. This dimerization induced an increase in the PRE effect for the KRAS: CRD interface within the KRAS: RBD–CRD complex, while the PRE pattern is preserved (Figure S19A, Supporting Information); however, no appreciable PRE change was observed for the adjacent KRAS‐RBD interface. These results demonstrated that the dimerization cooperatively stabilizes the interaction between KRAS and CRD, but not RBD, at the β‐interface in the KRAS: RBD–CRD complex.

**Figure 4:**
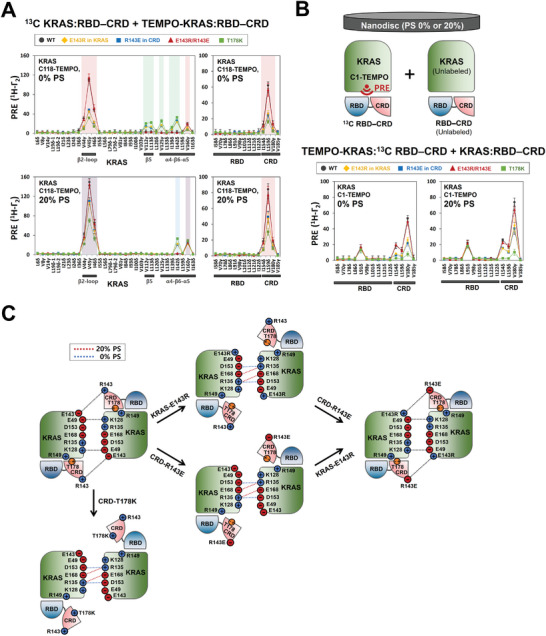
Mutagenic validation of key interactions of the CRD for dimerization of the heterodimeric KRAS: RBD–CRD complex. A) PRE rates for ILV ^13^C‐methyl probes in the KRAS: RBD–CRD complex and the interfacial mutants (E143R in KRAS, R143E in CRD, and E143R/R143E and T178K in CRD) in the presence of the same construct with a TEMPO spin label at Cys118 and the membrane lacking or containing 20% PS, as indicated. The PRE effects for effector‐free KRAS dimers on membranes lacking or containing 20% PS and those of the KRAS: RBD–CRD complex on both membranes are indicated by rectangular boxes shaded in green, blue, and red, respectively. Overlay of the PRE effects for free and effector‐bound KRAS dimers is shown in the box shaded in violet. B) Experimental design with ^13^C‐labeled RBD–CRD complexed with KRAS tagged with TEMPO at Cys1, isotopically unlabeled KRAS: RBD–CRD, and nanodisc, and PRE rates for ILV ^13^C‐methyl probes in the RBD–CRD in complex with membrane‐bound KRAS tagged with a TEMPO spin label at Cys1 near the β‐sheet effector binding site, and effects of dimerization of the KRAS: RBD–CRD complex or the interfacial mutants (E143R in KRAS, R143E in CRD, and E143R/R143E and T178K in CRD). C) A schematic of the effects of the interfacial single and double charge‐reversal mutations on intermolecular electrostatic interactions at the KRAS: KRAS and KRAS: CRD interfaces within the hetero‐tetrameric KRAS: RBD–CRD complex. Alternative electrostatic interactions of R135 with E168 or D153 at the KRAS dimer interface formed in the presence and absence of 20% PS are indicated by the red and blue dotted lines, respectively.

We further validated the electrostatic interaction between E143 of KRAS and R143 of the opposing CRD at the “α‐α” dimer interface of KRAS in our structural model. Consistently, charge‐reversal mutants, either KRAS‐E143R: RBD–CRD or KRAS: RBD–CRD‐R143E, exhibited a decrease in the PRE effects induced by the “α‐α” dimerization of the KRAS: RBD–CRD complex on both anionic and neutral membranes (**Figure** **4**A; Figure S20, Supporting Information). Concomitantly, the PRE effects, as observed for effector‐free dimers, were newly generated, indicating an equilibrium shift toward the dimer configuration adopted by effector‐free KRAS. The residual PRE of the KRAS: RBD–CRD dimer demonstrated that these mutations at the KRAS: CRD interface are not sufficient to fully disrupt the KRAS dimer bound to the RBD–CRD, in contrast to full inhibition of dimerization of the KRAS: RBD–CRD complex by both E49K and K128E mutations specific to the KRAS: KRAS interface (Figure 3C). The additional interaction of the CRD with a second KRAS protomer at the periphery of the KRAS: KRAS interface may play a supportive role in dimerization between two KRAS: RBD–CRD complexes. The reduced PRE effect was mitigated by mixing double charge‐reversal mutants, KRAS‐E143R: RBD–CRD and KRAS: RBD–CRD‐R143E, which is likely to restore the salt bridge at the KRAS: CRD interface to mediate dimerization of the KRAS: RBD–CRD complex. Notably, inhibition of the dimerization by both E143R and R143E mutants was concomitant with a large reduction in the intermolecular PRE effect between ^13^C‐labeled CRD and Cys1 TEMPO‐labeled KRAS in the preformed KRAS: RBD–CRD complex while the KRAS: RBD interface PRE is not affected (Figure 4B). The original PRE was recovered in the presence of KRAS‐E143R: RBD–CRD and KRAS: RBD–CRD‐R143E mutants, which were shown to favor dimerization. These mutations did not substantially impact the PRE induced by the KRAS: CRD interaction prior to the dimerization (Figure S19B, Supporting Information), indicating that reduced dimerization seen with both CRD‐R143E and KRAS‐E143R mutants is not the result of disrupting the preformed KRAS: CRD interface. Our mutagenesis data demonstrated that dimerization of the KRAS: RBD–CRD complex stabilizes the KRAS: CRD interface in this complex (Figure 4C).

We produced the CRD‐T178K mutant that is predicted by our model to inhibit the interaction between T178 in the CRD and R149 in KRAS in the KRAS: RBD–CRD complex (Figure 2). Compared to the wild‐type CRD, the T178K mutant indeed exhibited substantially diminishing PRE effects for probes in ^13^C‐labeled RBD–CRD upon binding to KRAS that is labeled with TEMPO at Cys1 near the β‐interface (Figure 4B). This result indicates that the T178K mutation promotes dissociation between KRAS and the CRD at the β‐interface. This mutation also induced largely reduced PRE effects for the “α‐α” dimer interface that mediates dimerization of the KRAS: RBD–CRD complex on both anionic and neutral membranes, concomitant with PRE increase for the effector‐free KRAS dimer (Figure 4A). This suggests that the KRAS: CRD interaction at the β‐interface in the KRAS: RBD–CRD complex promotes the signaling‐competent “α‐α” dimerization of this complex on the membrane. Consistently, the CRD T178A mutation, which is predicted in this study to destabilize the KRAS: CRD interface, was found to largely reduce KRAS‐dependent RAF kinase activity in mammalian cells.^[^ ^5g^ ^]^ The KRAS: CRD interaction may cause the proper orientation of a CRD interface to bind to a second KRAS molecule in complex with the RBD–CRD. Our mutagenic engineering of the KRAS: CRD interaction revealed mutual cooperativity between two KRAS: CRD interfaces in the hetero‐tetrameric KRAS: RBD–CRD complex.

### 2.4 Dimerization Alters Orientation of KRAS: RBD–CRD on the Membrane

**Figure 5:**
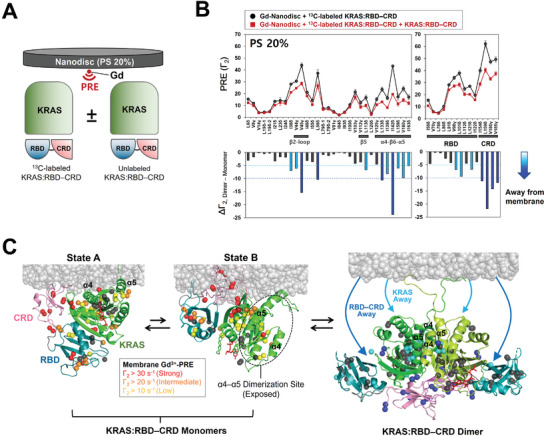
Dimerization‐induced changes in PRE effects on KRAS: RBD–CRD from spin labels on an anionic membrane surface containing 20% PS. A) Illustration of the experimental design with 100 µM [ILV‐^13^C methyl]‐labeled KRAS: RBD–CRD, 100 µM isotopically unlabeled KRAS: RBD–CRD, and a nanodisc (100 µM leaflet) containing 20% PS and a Gd^3+^spin label chelated to the headgroups of PE‐DTPA. B) ^1^H‐Γ_2_ PRE rates of ILV ^13^C‐methyl probes in KRAS: RBD–CRD induced by Gd^3+^‐associated membranes in the presence (red) and absence (black) of an equal amount of unlabeled KRAS: RBD–CRD, and changes in ^1^H‐Γ_2_ rates (Δ^1^H‐Γ_2,Dimer‐Monomer_) upon addition of KRAS: RBD–CRD (lower panel). Negative values of Δ^1^H‐Γ_2,Dimer‐Monomer_ represent loss of the membrane PRE effect upon dimerization. Probes with PRE changes are categorized according to the threshold values of Δ^1^H‐Γ_2,Dimer‐Monomer_: < −5 s^−1^ (cyan) and <−10 s^−1^ (blue). C) Structural models of the monomeric and dimeric states of the KRAS: RBD–CRD complex on anionic membranes containing 20% phosphatidylserine (PS). The monomer adopts two distinct orientational states, A and B, in equilibrium on the membrane. Dimerization promotes the release of the association of KRAS GTPase and RBD–CRD domains with the membrane, concomitant with interactions of the CRD with both KRAS protomers in the dimer. In the monomeric states, ^13^C probes are colored according to the threshold values of ^1^H‐Γ_2_: >10 s^−1^ (yellow), >20 s^−1^ (orange), and >30 s^−1^ (red). PRE‐unaffected probes are colored in dark gray. In the dimer, ^13^C probes are color‐coded according to the threshold values of Δ^1^H‐Γ_2,Dimer‐Monomer_ as in (B), and probes with no PRE changes are colored in dark gray.

Previous PRE and MD studies have revealed dynamic conformations of the KRAS: RBD–CRD complex on the membrane, in which CRD loosely associates with KRAS and engages the membrane containing PS.^[^ ^12^, ^13^ ^]^ To probe the dimerization‐induced change in orientation of the KRAS: RBD–CRD complex on the membrane, we performed PRE experiments using MSP1E3D1 nanodiscs containing a small amount of PE‐DTPA Nanodisc platforms and paramagnetic relaxation enhancement (PRE) analyses revealed that the binding of the RAS‐binding domain (RBD) generates two distinct KRAS dimer interfaces in equilibrium, favored by KRAS free and in complex with the RBD–cysteine‐rich domain (CRD) of RAF. Additional Interactions of the CRD with both KRAS protomers are mutually cooperative to stabilize a new dimer conformation of KRAS, which chelates paramagnetic Gd^3+^ ions on the lipid head. The ^1^H‐Γ_2_ values for the ILV ^13^C‐methyl groups in KRAS: RBD–CRD on the Gd^3+^‐associated nanodiscs were measured in the presence or absence of equivalent concentrations of isotopically unlabeled KRAS: RBD–CRD, which favor the dimeric and monomeric states of KRAS: RBD–CRD, respectively (**Figure** **5**; Figure S21, Supporting Information). Probes in the CRD of the monomeric state on the membrane containing 20% PS exhibited relatively large PRE effects, demonstrating that this domain binds or is at least transiently proximal to the membrane. Consistently, it has previously been revealed that two sites (residues F146–F151 and F158–G162) on the CRD are involved in the membrane association,^[^ ^14^ ^]^ which might stabilize the KRAS: RBD–CRD complex on the membrane. However, it appears that a single orientation of the monomeric KRAS: RBD–CRD complex on the membrane would not satisfy all the restraints derived from membrane Gd^3+^‐PRE effects (Figure 5B). To address this issue, we generated the HADDOCK structural models of the membrane‐bound KRAS: RBD–CRD monomer using PRE‐derived restraints between the proteins and membrane. A cluster analysis of these structures revealed two distinct orientational conformations in state A (major) and state B (minor) (Figure 5C; Figure S22, Supporting Information), which are essentially identical to the structures previously reported using the same approach (Figure S23, Supporting Information). Notably, both states commonly involve the interaction between the CRD and membrane. Additionally, the α4–α5 dimerization interface of KRAS in state A is occluded by the membrane, while state B exposes this interface to favor dimerization of the KRAS: RBD–CRD complex (Figure 5C).

The membrane PRE effects were substantially reduced by depleting PS in the membrane (Figure S21, Supporting Information), likely due to a lack of electrostatic interactions between KRAS: RBD–CRD and the membrane. Notably, the addition of unlabeled KRAS: RBD–CRD to favor dimerization induced overall reductions in the Gd^3+^‐PRE effects for ^13^C‐labeled KRAS: RBD–CRD on both membranes, indicating that dimerization leads the KRAS: RBD–CRD complex away from the membrane regardless of the PS content. Note that the absolute magnitude of PRE for KRAS: RBD–CRD residues closer to the Gd^3+^‐associated membrane is more sensitive to changes in their position. Even in the presence of the same equivalent of a second KRAS: RBD–CRD molecule, membrane Gd^3+^‐PRE effects arise from a mixture of the monomeric and dimeric states in equilibrium on the membrane. Consistently, the fraction of the KRAS: RBD–CRD dimer (F_dimer_) optimized while calculating the HADDOCK scores was 0.2–0.3 (Figure S14, Supporting Information). Thus, the membrane PRE effects observed in the presence of the second KRAS: RBD–CRD molecule (Figure 5B) may include signals from highly populated monomers on the membrane. Based on minimal PRE values (<10 s^−1^) for the dimeric state estimated from Equation S4 (Supporting Information), the reduction in overall PRE effects may reflect the population of dimers that have low PRE effects. The membrane PRE data obtained with the second KRAS: RBD–CRD molecule were incorporated to build the HADDOCK structural model of the membrane‐bound state of the KRAS: RBD–CRD dimer. The dimer structures converged well in a single cluster (Figure 5C; Figure S24, Supporting Information), with the KRAS GTPase (residues 1–172) and RBD–CRD domains far away from the membrane, while the disordered C‐terminal regions (residues 173–185) of KRAS protomers anchored the dimer to the membrane. This membrane orientation would be facilitated by the flexibility or release of interaction between the long‐disordered C‐terminal membrane anchor of KRAS and mobile lipids in the membrane. The HADDOCK structures of the monomeric and dimeric states of KRAS: RBD–CRD on the membrane suggest that dimerization promotes interactions of the CRD with both KRAS protomers in the dimer, concomitant with the dissociation of KRAS GTPase and RBD–CRD domains with the membrane.

### 2.5 Structural Mechanism for KRAS‐Mediated RAF Activation

**Figure 6:**
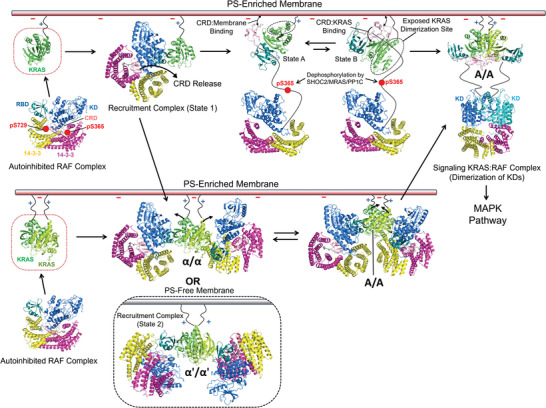
Structural model for the KRAS‐dependent activation process of RAF on a PS‐enriched membrane. In quiescent cells, RAF kinases exist in the cytosol and are inactivated by forming an autoinhibited complex with a 14‐3‐3 dimer, in which the 14‐3‐3 dimer binds serine phosphorylation sites (pS365 and pS729 in BRAF), restrains both the KD and CRD domains, and inhibits the functional dimerization of KDs. As an initial process in the RAF activation, the largely exposed RBD in this autoinhibited configuration binds to loosely associated monomers or transient dimers of KRAS in nanoclusters on a PS‐enriched lipid domain of the plasma membrane. This KRAS: RBD binding induces their full interaction to promote the formation of a “recruitment complex” of KRAS and RAF on the membrane while the CRD domain is buried at the center of the autoinhibited complex. The CRD is extracted from the recruitment complex and dynamically binds to both the anionic membrane and KRAS, which induces an “open” monomer conformation of RAF. This open state allows dephosphorylation of pS365 by the SHOC2 phosphatase complex and the 14‐3‐3 dimer rearrangement for the dimerization and activation of the KD. The KRAS: RBD–CRD complex undergoes two orientational conformations in equilibrium on a PS‐enriched membrane while the CRD is bound to the membrane: one exposes the KRAS dimerization interface whereas the other results in occlusion of this interface by the membrane. The membrane dissociation and KRAS binding of the CRD are promoted by dimerization of the KRAS: RBD–CRD complex, allowing additional binding of the CRD to the opposing KRAS protomer in this dimer. As an alternative pathway, the recruitment complex between KRAS and RAF monomers on a PS‐enriched membrane dimerizes through the KRAS: KRAS (indicated as α/α, R135‐E168) and KRAS: CRD interfaces to cooperatively form a hetero‐tetrameric KRAS: RAF complex on the membrane. This complex is also mediated by the binding of RAF to the preformed KRAS dimers in nanoclusters. PS depletion induces the formation of the recruitment complex with an alternative KRAS: KRAS interface (indicated as α’/α’, R135‐D153) while the KRAS: RBD interface is preserved. These PS‐modulated KRAS: KRAS interfaces are shifted toward a new dimer interface (indicated as A/A, E49‐K128) which provides an interaction site of KRAS for the opposing CRD. This process enhances cooperativity between the dimerization of the KRAS: RBD–CRD complex and the full interaction between KRAS and the RBD–CRD within this complex, resulting in the release and activation of the autoinhibited RAF. The structures in the RAF activation model are reconstituted based on our PRE‐derived structures of membrane‐bound KRAS dimers free and in complex with the RBD–CRD domain, the structures of the monomeric KRAS: RBD–CRD complex on the membrane (PDB IDs: 6PTS for state A and 6PTW for state B), and the cryo‐electron microscopy structures of the autoinhibited BRAF‐14‐3‐3 complex (PDB ID: 7MFE), the KRAS‐BRAF‐MEK‐14‐3‐3 recruitment complexes (PDB IDs: 8DGS for state 1 and 8DGT for state 2) and the active BRAF dimer in complex with MEK and 14‐3‐3 proteins (PDB ID: 6Q0J).

Membrane‐associated KRAS molecules are known to play a critical role in initiating the MAPK signaling cascade comprised of the RAF, MEK, and ERK kinases and promoting cell proliferation and oncogenesis.^[^ ^15^ ^]^ Notably, the mechanism for KRAS‐dependent activation of RAF kinases at the plasma membrane is tightly regulated, compared to less complex activation mechanisms of MEK and ERK kinases,^[^ ^16^ ^]^ which commonly involve a conformational change to open the catalytic site for substrate phosphorylation. Recent studies have determined the crystal structures of RAS monomers in complex with the RBD–CRD of RAF and the cryo‐electron microscopy (cryo‐EM) structures of the autoinhibited complex and active KD dimer of full‐length RAF with a 14‐3‐3 dimer[5a‐d, 5 g], significantly advancing our understanding of the regulatory mechanisms for RAF activation. These structural snapshots and effector‐modulated modes of KRAS dimerization, presented here, are combined to provide integrated structural models for KRAS‐dependent RAF activation through multistep processes including dynamic configurations of KRAS dimers on the membrane (**Figure** **6**). All RAF family members, RAF1/CRAF, ARAF, and BRAF, have an N‐terminal regulatory domain that contains the RBD followed by the CRD and a flexible linker region possessing S365 (residue number in BRAF), a phosphorylation site for binding to a 14‐3‐3 scaffold, and a C‐terminal KD that can dimerize to function. In resting cells, RAF monomers localize to the cytosol and exist as a closed, autoinhibited configuration including interactions between the RBD–CRD, KD, phosphorylated sites on linker regions, and a 14‐3‐3 dimer.

KRAS forms nanoclusters, potentially comprising KRAS dimers, higher‐order oligomers, and even loosely associated monomers in proximity, primarily on PS‐enriched lipid domains of the plasma membrane. PS interacts with the polybasic region at the C‐terminal membrane anchor of KRAS. Nanoclusters have been proposed to be transiently formed in a switch‐like manner and act as essential units for recruiting and activating downstream RAF effectors.^[^ ^10^, ^17^ ^]^ Membrane‐bound KRAS monomers initially bind to the RBD that is largely exposed in the autoinhibited complex, but not the CRD that is buried at the center of the complex. This KRAS: RBD binding induces an intermediate “recruitment complex” of RAF on the membrane. To fully release this RAF complex, the CRD is released and dynamically binds to the anionic membrane and/or a β‐sheet region adjacent to the RBD binding site of KRAS in the preformed KRAS: RBD complex. The KRAS: RBD–CRD complex undergoes two orientational conformations in equilibrium on an anionic membrane containing PS while the CRD is bound to it.^[^ ^12^ ^]^ There are two possible conformations: one exposes the KRAS dimerization interface that can then bind to a second KRAS molecule whereas the other results in occlusion of this interface by the membrane. The membrane dissociation of the CRD and the interaction between the released CRD and KRAS are promoted by dimerization of the KRAS: RBD–CRD complex, allowing additional binding of the CRD to the opposing KRAS protomer in this dimer.

An alternative mechanistic model is that the RBD in the autoinhibited complex binds to KRAS dimers. Previous quantitative fluorescence data have revealed that monomers are the predominant form of KRAS at the physiological expression level, with dimers being more prevalent than other higher‐order oligomers.^[^ ^4a^ ^]^ The binding of the RBD to KRAS protomers on both anionic and neutral membranes promotes an alternative KRAS dimer interface, which is favored by KRAS dimers when bound to the RBD–CRD. The formation of the hetero‐tetrameric KRAS: RBD–CRD complex is facilitated by the conformational selection mechanism involving the preformed alternative KRAS dimer in complex with the RBD alone, which is poised to recruit the CRD. The CRD becomes highly stabilized by engaging both protomers in the KRAS dimer with the RBD–CRD domain, thereby accelerating the release of the autoinhibited state of RAF.

Both mechanistic scenarios synergistically result in the formation of a signaling‐competent KRAS: RAF complex in which the KD of RAF dimerizes to function on a 14‐3‐3 dimer cradle, as well as the exposure of the autoinhibitory phosphorylation site (S365) at a flexible linker between the CRD and KD. This residue can be dephosphorylated by the SHOC2/MRAS/PP1C complex to maintain the activated configuration of the KRAS: RAF complex in the RAF activation cycle. In the absence of PS, which promotes KRAS self‐assembly,^[^ ^10^ ^]^ the RAF activation is initiated by the KRAS: RAF hetero‐dimerization prior to KRAS homodimerization, rather than the nanocluster‐stimulated RAF activation. Furthermore, the assembly process for a signaling‐competent hetero‐tetrameric complex comprising KRAS and RAF dimers may be affected by the relative stoichiometry between RAS, RAF, and other RAS effectors and regulators, which varies in many types of normal or KRAS‐driven cancer cells.^[^ ^18^ ^]^ It has been argued that KRAS nanoclusters represent proximal monomers simply because of their recruitment by KRAS‐lipid interactions with specific size‐limited domains on the membrane.^[^ ^19^ ^]^ However, PRE and mutagenesis studies have demonstrated that KRAS self‐assembly involves physical interaction between the catalytic GTPase domains of KRAS.^[^ ^5^, ^20^ ^]^ It remains undetermined whether all RAF isoforms have a similar autoinhibited conformation, whether the functional dimerization of KDs in the cytosol impacts the dimerization mode of KRAS in complex with the RBD–CRD on the membrane, and why the heterodimeric RAF1‐BRAF complex predominates in the RAS‐driven signaling pathway.

## 3 Conclusion

Membrane‐anchored KRAS molecules self‐associate to act as scaffolds for promoting the functional dimerization of downstream RAF kinases, which are implicated in a quarter of all human cancers. Bindings of the N‐terminal RBD or RBD–CRD domains of RAF1 differentially alter the dimerization mode of KRAS on both anionic and neutral membranes. Notably, the dynamic CRD binding is a key determinant of the relative orientation between two protomers in the KRAS dimer in complex with to the RBD–CRD. The KRAS: CRD interaction and KRAS dimerization are mutually cooperative to stabilize a hetero‐tetrameric KRAS: RAF complex that mediates the MAPK signaling pathway. Assembling structures of KRAS and RAF provides the mechanistic model for KRAS‐dependent RAF activation where initial binding of the RBD in the autoinhibited RAF complex rearranges the interprotomer orientation of the KRAS dimer to promote the KRAS: CRD interaction and release the autoinhibited state. The effector domain‐specific modulation of KRAS dimerization provides insights into novel therapeutic inhibition of a signaling KRAS: RAF complex. Furthermore, our PRE‐based methodology will be applied to many other signaling protein complexes on the membrane.

## Conflict of Interest

The authors declare no conflict of interest.

## Supporting information

Supporting Information

## Data Availability

The data that support the findings of this study are available from the corresponding author upon reasonable request.
