# Supplementary material for: Effector Binding Sequentially Alters KRAS Dimerization on the Membrane: New Insights Into RAS‐Mediated RAF Activation
Source: Adv Sci (Weinh). 2024 Aug 13;11(38):2401530. doi: 10.1002/advs.202401530 (PMC11481233; doi:10.1002/advs.202401530)
Supplement: Supplementary file 1 — Supporting Information [file ADVS-11-2401530-s001.docx]

**Effector Binding Sequentially Alters KRAS Dimerization on the Membrane: New Insights Into RAS-Mediated RAF Activation**

**Table of Contents**

1. Material and Methods …………………………………….…………………….……… 2

2. Supplementary Figures …………………..…………………….…...….……………... 8

3. Supplementary Tables ………….…………………………………………………..…. 37

4. References …………………………..……………………………….……..….……… 47

**1. Material and Methods**

**1.1. Protein Preparation**

Native, fully processed KRAS, in which the C-terminal Cys185 is farnesylated and methylated, was obtained from the baculovirus-insect cell (Hi5) expression system, as described previously(1). To isotopically label KRAS with U-^13^C-[Ile, Leu, Val], Hi5 insect cells were grown in complete insect cell medium (ESF 921, Expression Systems Inc.) to a cell density of 1.5 x 10^6^ cells/mL. The culture was then harvested and resuspended in customized ESF921 medium lacking Ile, Leu, and Val. The resuspended cells were infected with baculovirus containing the KRAS gene, grown for 16 h, then supplemented with 150 mg/L ^13^C-Ile, 100 mg/L ^13^C-Leu, and 50 mg/L ^13^C-Val. For nucleotide exchange, GDP-loaded KRAS was incubated overnight at 4°C with a 20-fold molar excess of GTPγS (guanosine 5′-[γ-thio]triphosphate tetralithium salt, Sigma-Aldrich) and 10 mM EDTA, followed by size exclusion chromatography (Superdex 75; Cytiva). The RAS binding domain (RBD) (residues 53-137) of RAF1 was expressed as a GST fusion from pGEX4T2, and the RBD–cysteine rich domain (CRD) (55-187) of RAF1 was expressed as a His-tagged fusion from pET28a. *Escherichia coli* was grown either in Luria broth (LB) to produce unlabeled protein or minimal M9 media supplemented with 60 mg/mL 2-ketobutyric acid-4-^13^C and 100 mg/mL of 2-keto-3-(methyl-^13^C)-butyric acid to ^13^C label the methyl groups of the Ile-Cδ1, and Leu-Cδ and Val-Cγ, respectively. Protein expression was induced with 0.2 mM isopropyl-1-thio-β-D galactopyranoside (IPTG) at 15 °C overnight, and 10 µM ZnCl_2_ was added to the culture media and purification buffers when the RBD–CRD protein was induced. Proteins were purified using Ni^2+^-NTA or Glutathione Sepharose resin, followed by thrombin digestion overnight at room temperature to remove His or GST tags. The thrombin-cleaved proteins were further purified by size exclusion chromatography using Superdex-75 columns (Cytiva)(2, 3). Single-cysteine mutations (M1C and K169C) and were introduced into KRAS C118S using a standard PCR mutagenesis protocol. Likewise, single or double charge-reversal interface mutations (E49K, K128D, E143R, D153K, E168R, and E49K/K128D) were introduced into both ^13^C-labeled and unlabeled KRAS molecules, and the T178K or R143E mutation was introduced into both ^13^C-labeled and unlabeled CRDs of RAF1.

**1.2. Nanodisc Preparation**

Lipid bilayer nanodiscs encircled by two copies of membrane scaffold protein 1E3D1 (MSP1E3D1) were prepared using a 1:40 MSP1E3D1:lipid molar ratio. A 2 mM stock solution of MSP1E3D1 was kept at -80 °C. Since MSP proteins are prone to self-association and invisible aggregation in solution, the molar ratio of lipid to the active protein should be higher than 40. This nanodisc provides the lipid surface with a diameter of ~11 nM, which can accommodate a maximum of seven KRAS proteins (~36 Å each), three or four KRAS:RBD complexes (~45 Å each), or three KRAS:RBD–CRD complexes (~55 Å each). A molecular simulation method (http://www.charmm-gui.org/input/nanodisc) was used to build the structural model for the MSP1E3D1-type nanodisc, which contains 27 DOPS (20%) and 109 DOPC (80%) lipids per leaflet. Since the assembled nanodisc contains 27 DOPS and 109 DOPC lipids per leaflet, they provide ~7 PS lipids to interact with each protomer in the KRAS:RBD–CRD dimer.

All lipids were purchased from Avanti Polar Lipids, Inc., including 1,2-dioleoyl-sn-glycero-3-phosphocholine (DOPC), 1,2-dioleoyl-sn-glycero-3-phospho-L-serine (DOPS), and 1,2-distearoyl-snglycero-3-phosphoethanolamine-N-diethylenetriaminepentaacetic acid (PE-DTPA). DOPC and DOPS lipids were mixed at molar ratios of 8:2 to produce nanodiscs containing 20% phosphatidylserine (PS) to mimic the inner leaflet of the plasma membrane. To produce nanodiscs lacking PS, DOPS lipids were replaced with DOPC lipids. To produce paramagnetic Gd^3+^-associated nanodiscs containing or lacking 20% DOPS, DOPC, DOPS, and PE-DTPA lipids were mixed at molar ratios of 75:20:5 and 95:0:5, respectively. MSP1E3D1 was expressed in *Escherichia coli* BL21 (DE3) from the pGBHPS-MSP vector in 2× yeast extract tryptone media using a LEX bioreactor system. Expression was induced at 37 °C with 1 mM IPTG at an OD_600_ of 2.5 for 1 h, followed by further incubation at 28 °C for 2.5 h. The protein was purified using a Ni^2+^-NTA column, followed by cleavage of the His_6_-GB1 tag with HRV3C protease, and final purification by size exclusion chromatography (Superdex 200; Cytiva).

**1.3. NMR Experiments**

NMR experiments were carried out at 288 K on a Bruker AVANCE III HD 800-MHz spectrometer equipped with 5-mm TCI CryoProbe. All NMR samples were prepared in 50 mM HEPES pH 7.4, 300 mM NaCl, 5 mM MgCl_2_, 10% (vol/vol) D_2_O, 10 μM ZnCl2, and 5 mM GTPγS. For TEMPO-PRE experiments, two-dimensional (2D) ^1^H-^13^C TROSY[1] were collected from 100 μM ^13^C-labeled KRAS free or in complex with RAF1 domains, the RAS binding domain (RBD) or RBD-cysteine rich domain (CRD), in the presence of 100 μM of the same construct tagged with TEMPO spin labels. To reduce the number of overlapping peaks for the ILV ^13^C-methyl probes in the NMR spectra, NMR experiments were performed using two separate samples: ^13^C-labeled KRAS in complexes with isotopically unlabeled RBD or RBD–CRD and isotopically unlabeled KRAS in complexes with ^13^C-labeled RBD or RBD–CRD. Two molar ratios of KRAS, KRAS–RBD, or KRAS:RBD–CRD to the nanodisc leaflets (100 μM) were used to promote their dimerization on the membrane. To perform membrane PRE experiments, 100 μM nanodisc leaflets containing 5% Gd^3+^-chelated PE-DTPA were mixed with 100 μM ^13^C-labeled KRAS:RBD–CRD or 100 μM ^13^C-labeled KRAS:RBD–CRD and 100 μM isotopically unlabeled KRAS:RBD–CRD. The relaxation delay of 8 s used in the PRE experiments was relatively long compared to those commonly used (1–1.5 s) in protein NMR experiments. A relaxation delay more than four times longer than the longitudinal relaxation time (T1) would be required for equal recovery of the longitudinal magnetization for diamagnetic and paramagnetic samples to enable accurate measurement of the PRE rates (^1^H-Γ_2_)[2]. It was estimated that the longitudinal relaxation times (T1) of KRAS, KRAS:RBD, and KRAS:RBD–CRD with molecular weights of ~22–37 kDa would be 1–1.5 s under our experimental conditions, and that freely diffusing monomers in solution and on the membrane surface would be the predominant forms (~75–90%). TEMPO spin labels (Toronto Research Chemicals) were attached to the thiol group of one of three single free cysteines (Cys1, Cys118, or Cys169) of KRAS. A concentrated stock of TEMPO in acetonitrile was added at a 5:1 TEMPO:KRAS molar ratio, and the mixture was incubated at 4°C for 16 h, then dialyzed at 4°C for 8 h into NMR buffer to remove the residual TEMPO reagent. For diamagnetic control experiments, the TEMPO spin label (100 μM) was reduced with 1 mM ascorbic acid at 15°C for at least 2 h to ensure complete reduction. Lu^3+^-chelated PE-DTPA was used as a diamagnetic control to replace paramagnetic Gd^3+^-chelated PE-DTPA. The peaks were assigned based on assignments deposited in the Biological Magnetic Resonance Data Bank (BMRB) by our group and others (entry number: 17610, 17785, 25115, 27472, 30401, and 30639)

**1.4. PRE Analysis**

NMR data were processed and analyzed using NMRPipe[3] and NMRView[4]. The intensities of the cross-peaks were measured with Lorentzian line shape fitting using the program nlinLS in the NMRPipe software package[3]. The transverse relaxation rate in the diamagnetic state, R_2_, was estimated from the half-height line width of each peak as described by Wagner and co-workers[5]. Overlapped cross-peaks in the spectra were excluded from the analysis. In TEMPO-PRE experiments, peak intensities were referenced to the ^1^H peak intensity of 4,4-dimethyl-4-silapentane-1-sulfonic acid (DSS) although paired spectra were obtained using identical sample concentrations and NMR acquisition parameters. PRE effects on individual probes of the KRAS:RBD–CRD complex were assessed by the ratio of their peak intensities in the paramagnetic versus diamagnetic samples (I_para_/I_dia_). These values were converted to ^1^H transverse PRE rates (^1^H-Γ_2_) using equation 1:

$\frac{I_{\mathrm{para}}}{I_{\mathrm{dia}}}=\frac{R_{2}\times exp (-\Gamma_{2}\times t)}{R_{2}+ \Gamma_{2}}$ (equation 1)

where t is the total INEPT evolution time (7.2 ms) in the NMR pulse sequence. The PRE effect on ^1^H longitudinal relaxation rate (^1^H-R_1_) is typically negligible because it is much smaller than ^1^H-Γ_2_. The paramagnetic relaxations of ^15^N and ^13^C nuclei were ignored in the analysis because they have much lower gyromagnetic ratios than ^1^H nucleus. Errors in the intensity ratios between paramagnetic and diamagnetic peaks [∆(I_para_/I_dia_)] on the spectra were estimated using equation 2:

$\Delta(I_{\mathrm{para}}/I_{\mathrm{dia}})=\sqrt{({\Delta I_{\mathrm{para}}/I_{\mathrm{para}})}^{2}+ ({\Delta I_{\mathrm{dia}}/I_{\mathrm{dia}})}^{2}}$ (equation 2)

where ∆I_para_ and ∆I_dia_ are noise levels of I_para_ and I_dia_, respectively, on the spectra measured using NMRPipe[3]. The ^1^H-Γ_2_ values were converted into distances (r) between observed protons and the paramagnetic spin label using equation 3:

$r= \sqrt[6]{\frac{F_{\mathrm{dimer}}}{\Gamma_{2}}\frac{\gamma_{H}^{2}g_{e}^{2}\beta^{2}u_{0}^{2}\left( S+1 \right)S}{240\pi^{2}}(4\tau_{c}+\frac{3\tau_{c}}{1+\omega_{H}^{2}\tau_{c}^{2}})}$ (equation 3)

where F_dimer_ is the fraction of the dimer of the KRAS:RBD–CRD complex, γ_H_ is the proton nuclear gyromagnetic ratio, g_e_ is the electronic g factor, β is the Bohr magneton, u_0_ is the vacuum permeability, S is the spin quantum number for free electrons, 𝜔_H_ is the proton Larmor frequency, and τ_c_ is the rotational correlation time of the electron-nucleus vector. τ_c_ was assumed to be equal to the global correlation time of the complex. The molecular weight of the complex between the KRAS:RBD–CRD dimer and MSP1E3D1 nanodisc was used to provide an estimated τ_c_ value of 193 ns. Note that the calculated distance is insensitive to large errors in τ_c_ due to r^-6^ dependence. The error bars in the ^1^H-Γ_2_ plots were generated from errors in the intensity ratios between paramagnetic and diamagnetic peaks [∆(I_para_/I_dia_)] on the spectra. In the analysis of the membrane PRE data, net ^1^H-Γ_2_ values of the KRAS:RBD–CRD dimer can be extracted from a mixture of ^1^H-Γ_2_ of the monomeric and dimeric species with the following equation 4, assuming that the monomer-dimer equilibrium is in the fast exchange regime on the Γ_2_ relaxation time scale.

^1^H-Γ_2_,_app_ ≈ F_mono_ x ^1^H-Γ_2_,_mono_ + F_dimer_ x ^1^H-Γ_2_,_dimer_ (equation 4)

where ^1^H-Γ_2,app_ is the population-weighted average of ^1^H-Γ_2,mono_ and ^1^H-Γ_2,dimer_, which represent ^1^H-Γ_2_ values for the monomeric and dimeric states, respectively, and F_mono_ and F_dimer_ represent the relative fractions of the monomeric and dimeric states, respectively.

**1.5. Building Structures of the Hetero-Tetrameric KRAS:RBD–CRD Complex**

The standard protocol embedded in the Haddock 2.2 program[6] was used to generate the structural models of the membrane-bound KRAS dimer of which protomers bind to the tandem RBD–CRD domain of RAF1. Unambiguous distance restraints for defining the dimer interface between two KRAS protomers bound to the RBD–CRD were derived from the TEMPO-PRE data of the KRAS:RBD–CRD complex that exhibit ^1^H-Γ_2_ values > 10 s^-1^. Distances from PRE-observed carbon atoms to the spin-labeled Cys gamma positions of KRAS were used in a symmetric manner for the two KRAS protomers. Variable sets of distance restraints were calculated by changing the fraction of observed KRAS:RBD–CRD sample (F_dimer_) bound to the paramagnetic KRAS:RBD–CRD in equation 2. Goodness-of-fit of distances were assessed by the standard distance restraint potential, E_noe_. The lowest E_noe_ values were found with a F_dimer_ of 0.25. The optimized F_dimer_ value for the KRAS:RBD–CRD dimer (0.25) indicates low dimerization efficiency, which may not favor the potential formation of higher-order oligomers from dimers. Thus, the majority of KRAS molecules are likely to be monomers in the free or membrane-bound state. To account for errors in the estimate of ^1^H-Γ_2_, distance restraint bounds were set to ±3 Å from the calculated distance. The r^-6^ dependency of ^1^H-Γ_2_ markedly reduces the uncertainty of distances in the PRE analysis. Distance restraints between the KRAS:RBD–CRD complex and lipid headgroups on one leaflet of the nanodisc were derived from the membrane PRE data obtained with and without equivalent amounts of the same equivalent of a second, unlabeled KRAS:RBD–CRD molecule. To build the Haddock structural model of the monomeric KRAS:RBD–CRD complex on the membrane, ambiguous interaction restraints (AIRs) between the nanodisc surface and ^13^C probes with the ^1^H-Γ_2_ value of >20 s^-1^ were set to be a range of 2–5 Å. Based on the F_dimer_ of 0.2–0.3 optimized during the HADDOCK structure calculation, overall ^1^H-Γ_2,dimer_ values were estimated to be minimal (<10 s^-1^). In the Haddock calculation of the KRAS:RBD–CRD dimer on the membrane, ^13^C probes that exhibit measurable reductions in the membrane PRE effect caused by the addition of a second KRAS:RBD–CRD molecule (Δ^1^H-Γ_2_,_dimer-mono_ of <-10 s^-1^) were set to have the upper limits (>10 Å), which correspond to the ^1^H-Γ_2,dimer_ value of <10 s^-1^ induced by the membrane containing 5% Gd^3+^ ions. To maintain membrane association of the KRAS C-terminus, an upper limit of 2 Å was set as an ambiguous restraint for Lys184. The NMR-driven structure of the monomeric KRAS:RBD–CRD complex (PDB ID: 6PTS) was used as a starting model to determine the structures of its membrane-bound states, and the crystal structure of the KRAS:RBD–CRD heterodimer (PDB ID: 6xi7) was used as a starting model to construct the hetero-tetrameric KRAS:RBD–CRD complex comprising two KRAS:RBD–CRD heterodimers. According to the sampling protocol, 1000 complex structures were generated in the first step of the rigid body docking, 200 structures were selected in the semi-flexible docking, and 20 structures were finally submitted to the water refinement steps. Violation analysis of PRE-derived distances in the final Haddock model was performed using a modified Q-factor[7], a value that represents the agreement between distances observed in PRE experiments (r^obs^) versus the Haddock model distances (r^model^), defined as:

Q-factor =$\sqrt{\frac{\sum_{i} \{\text{r}\text{obs} \left( i \right) - \text{r}\text{model }\text{(}i\text{)\}}\text{2}}{\sum_{i} \text{r}\text{obs}\text{(}i\text{)}\text{2}}}$ (equation 5)


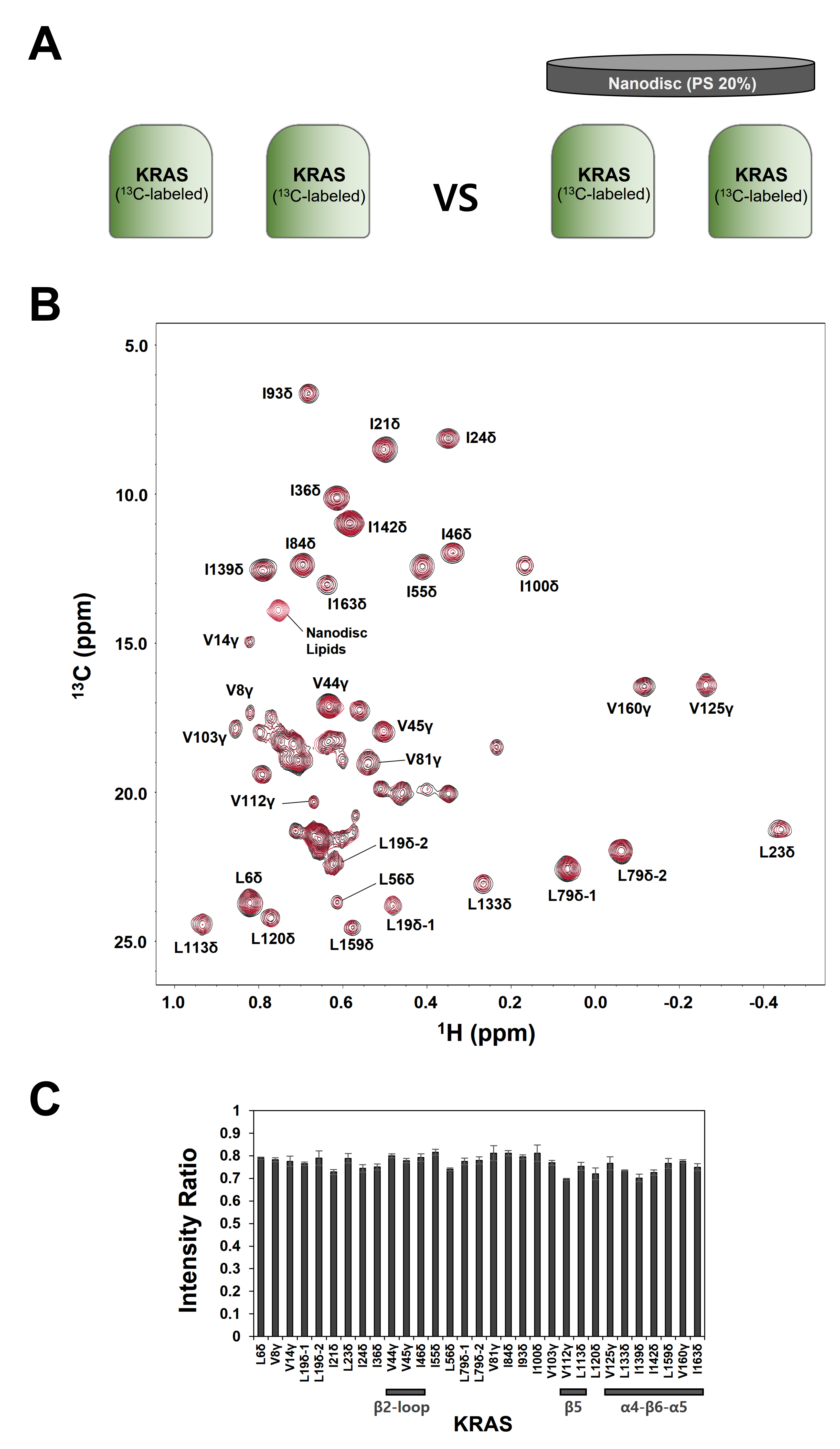
**2. Supporting Figures**

**Figure S1.** No large differential reductions in peak intensities for KRAS upon the addition of nanodiscs. (A) A schematic of the NMR experimental design with 200 uM [ILV-^13^C methyl]-labeled KRAS in the presence of 100 uM lipid leaflets of MSP1E3D1 nanodiscs containing PS. (B) Overlaid ^1^H-^13^C TROSY spectra for ^13^C-labeled KRAS in the presence (red) and absence (black) of nanodiscs. (C) Plot of the intensity ratios of the peaks for ILV ^13^C-methyl probes in the spectra in panel (B).


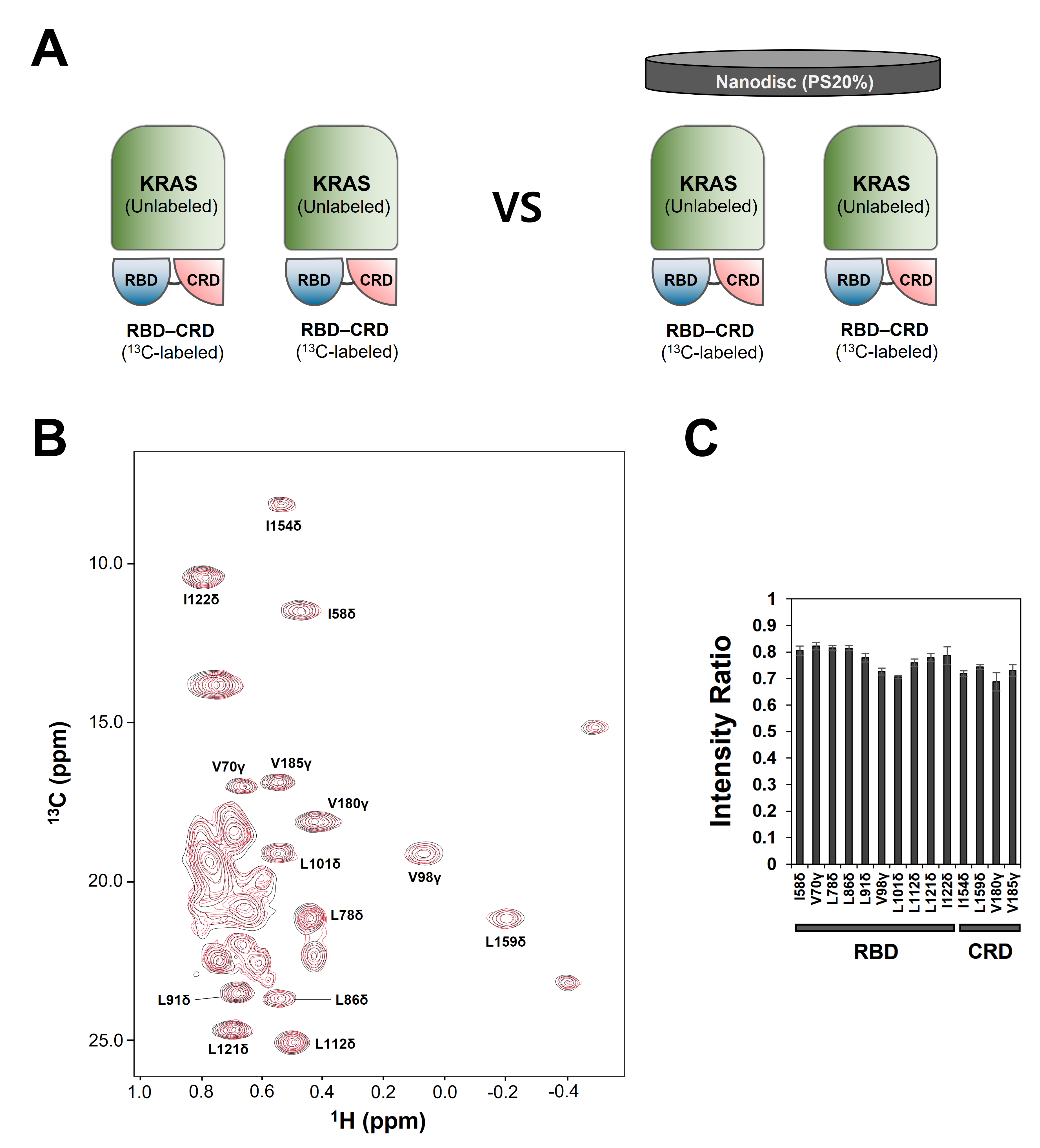


**Figure S2.** No large differential reductions in peak intensities for the RBD–CRD in complex with KRAS upon the addition of nanodiscs. (A) A schematic of the NMR experimental design with 200 uM [ILV-^13^C methyl]-labeled RBD–CRD bound to isotopically unlabeled KRAS (KRAS:^13^C RBD–CRD) in the presence of 100 uM lipid leaflets of MSP1E3D1 nanodiscs containing 20% PS. (B) Overlaid ^1^H-^13^C TROSY spectra for KRAS:^13^C RBD–CRD in the presence (red) and absence (black) of nanodiscs. (C) Plots of the intensity ratios of the peaks for ILV ^13^C-methyl probes in the spectra in panel (B).


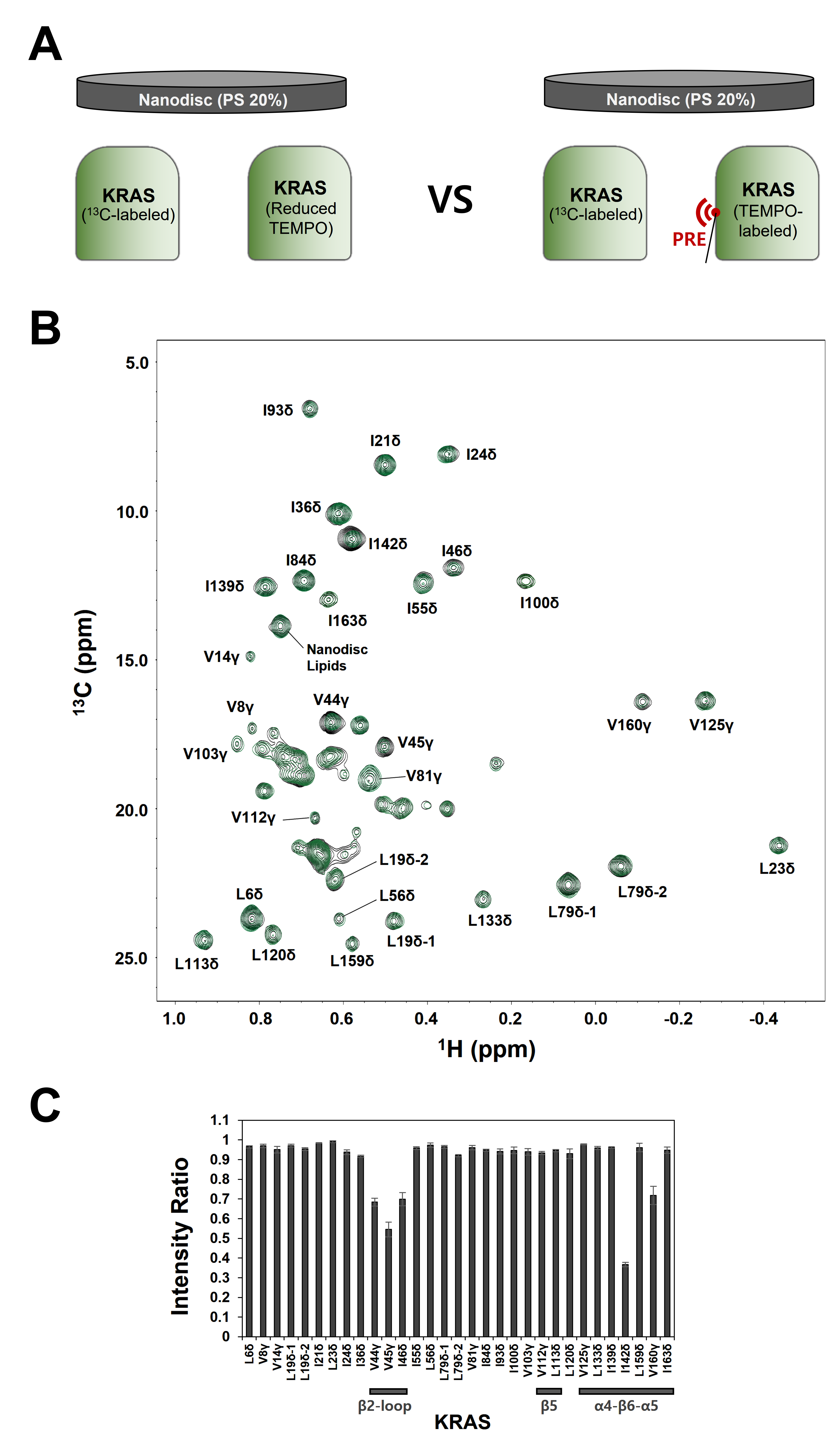


**C118-TEMPO**

**Figure S3.** PRE-induced reductions in peak intensities for KRAS upon dimerization on the membrane. (A) A schematic of the PRE experimental design with 100 uM [ILV-^13^C methyl]-labeled KRAS and 100 uM KRAS tagged with TEMPO at Cys118 in the presence of 100 uM lipid leaflets of MSP1E3D1 nanodiscs containing 20% PS. (B) Overlaid ^1^H-^13^C TROSY spectra for ^13^C-labeled KRAS in the presence (green) and absence (black) of a PRE spin at Cys118 of a second KRAS molecule. (C) Plots of the intensity ratios of the peaks for ILV ^13^C-methyl probes in the spectra in panel (B).


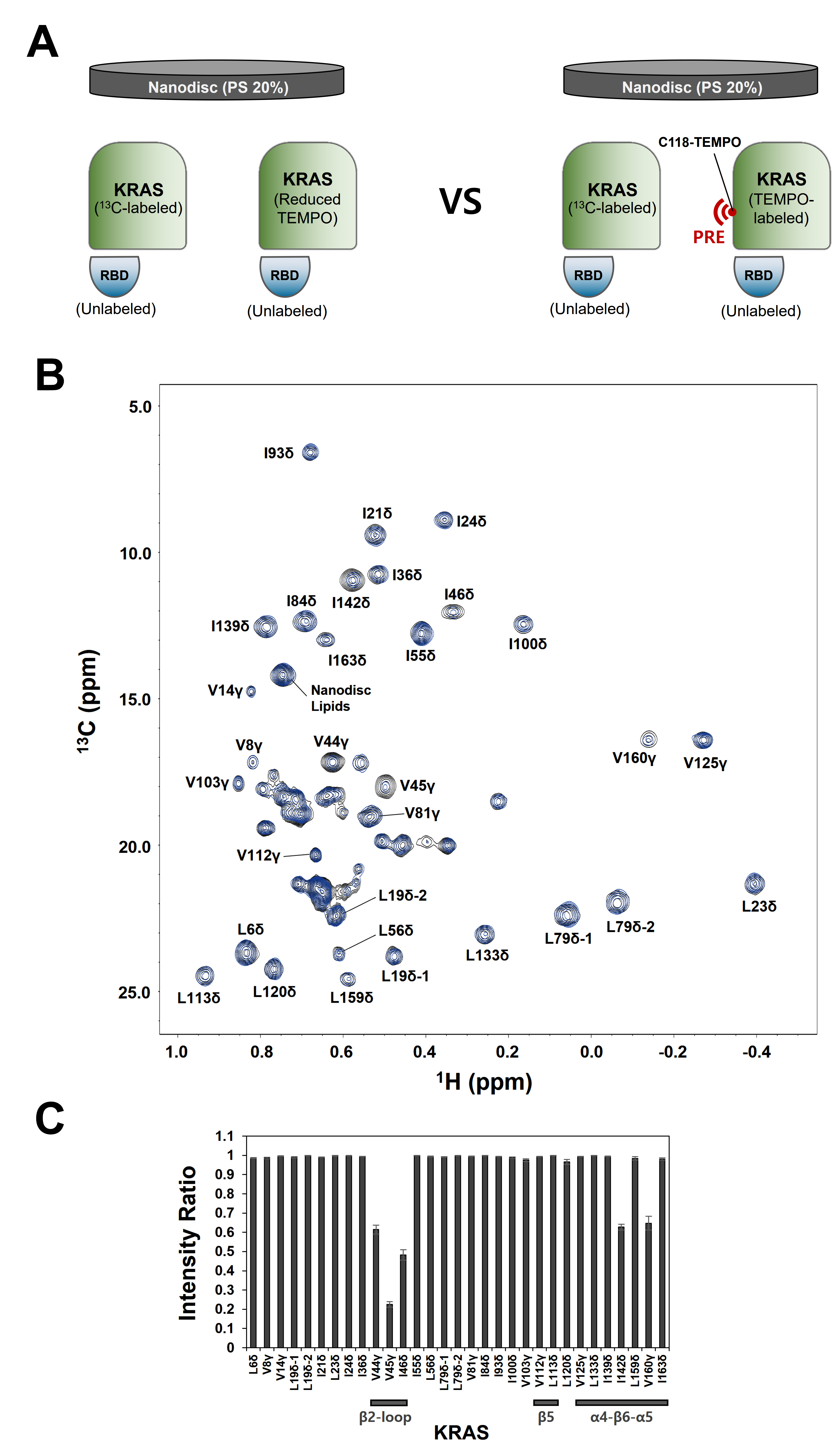


**Figure S4.** PRE-induced reductions in peak intensities for KRAS in complex with RBD upon dimerization on the membrane. (A) A schematic of the PRE experimental design with 100 uM [ILV-^13^C methyl]-labeled KRAS complexed with the isotopically unlabeled RBD (^13^C KRAS:RBD) and 100 uM KRAS:RBD tagged with TEMPO at Cys118 of KRAS in the presence of 100 uM lipid leaflets of MSP1E3D1 nanodiscs containing 20% PS. (B) Overlaid ^1^H-^13^C TROSY spectra for ^13^C KRAS:RBD in the presence (blue) and absence (black) of a PRE spin at Cys118 of KRAS in complex with RBD. (C) Plots of the intensity ratios of the peaks for ILV ^13^C-methyl probes in the spectra in panel (B).


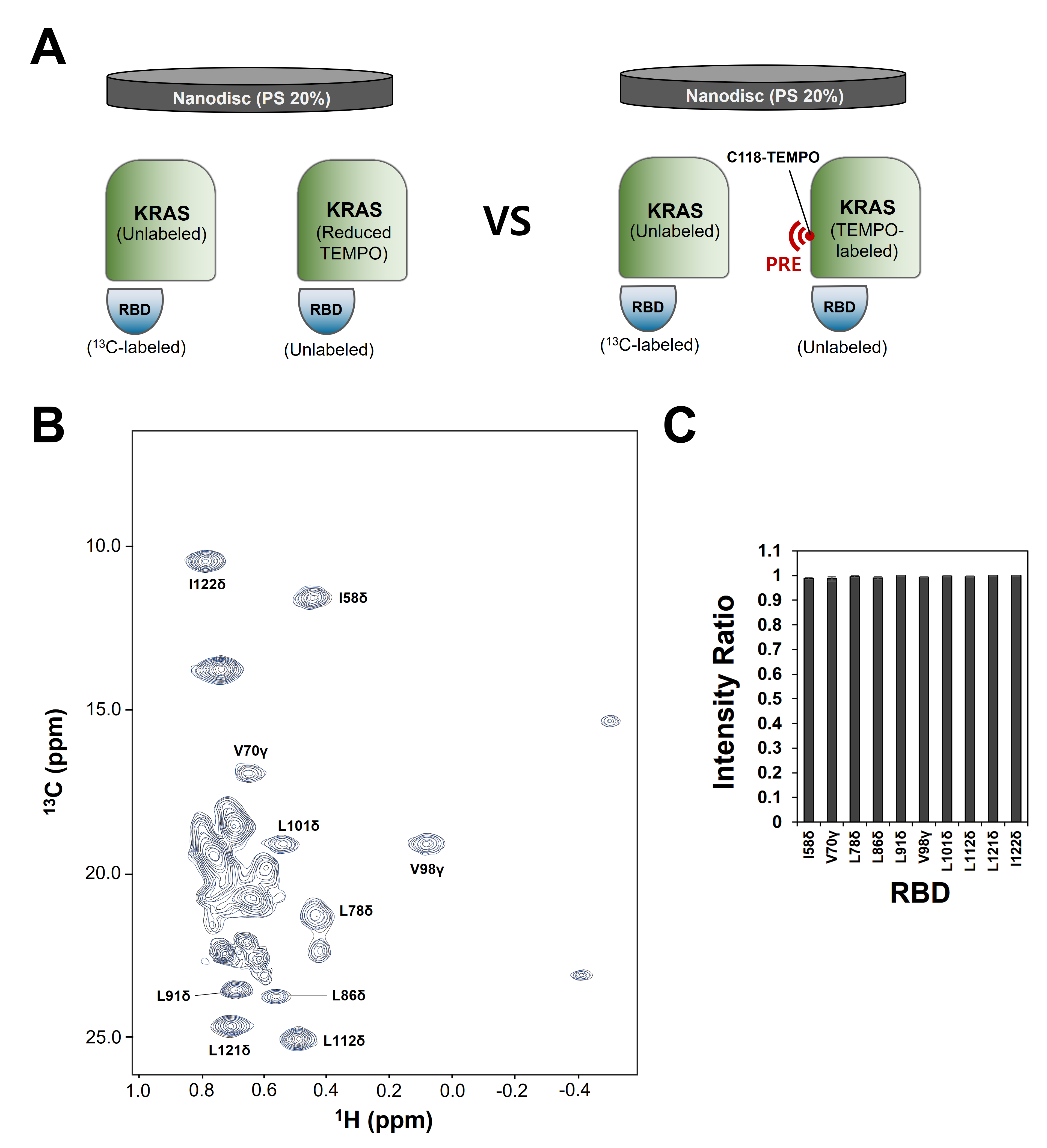


**Figure S5.** No PRE-induced reductions in peak intensities for RBD in complex with KRAS upon dimerization on the membrane. (A) A schematic of the PRE experimental design with 100 uM [ILV-^13^C methyl]-labeled RBD complexed with isotopically unlabeled KRAS (KRAS: ^13^C RBD) and 100 uM KRAS:RBD tagged with TEMPO at Cys118 of KRAS in the presence of 100 uM lipid leaflets of MSP1E3D1 nanodiscs containing 20% PS. (B) Overlaid ^1^H-^13^C TROSY spectra for KRAS:^13^C RBD in the presence (blue) and absence (black) of a PRE spin at Cys118 of KRAS in complex with RBD. (C) Plots of the intensity ratios of the peaks for ILV ^13^C-methyl probes in the spectra in panel (B).


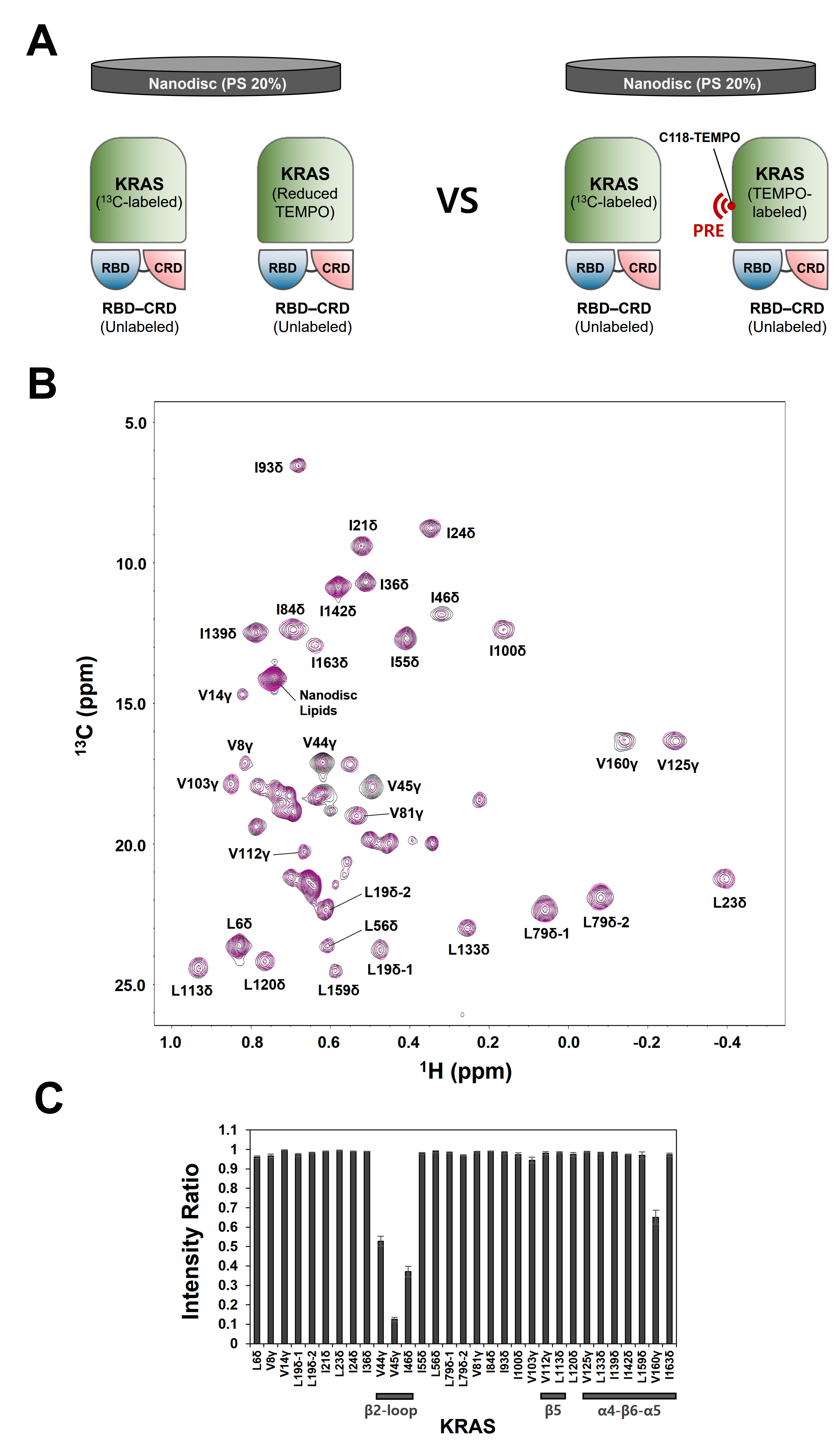


**Figure S6.** PRE-induced reductions in peak intensities for KRAS in complex with RBD–CRD upon dimerization on the membrane. (A) A schematic of the PRE experimental design with 100 uM [ILV-^13^C methyl]-labeled KRAS complexed with the isotopically unlabeled RBD–CRD (^13^C KRAS:RBD–CRD) and 100 uM KRAS:RBD–CRD tagged with TEMPO at Cys118 of KRAS in the presence of 100 uM lipid leaflets of MSP1E3D1 nanodiscs containing 20% PS. (B) Overlaid ^1^H-^13^C TROSY spectra for ^13^C KRAS:RBD–CRD in the presence (violet) and absence (black) of a PRE spin at Cys118 of KRAS in complex with RBD–CRD. (C) Plots of the intensity ratios of the peaks for ILV ^13^C-methyl probes in the spectra in panel (B).


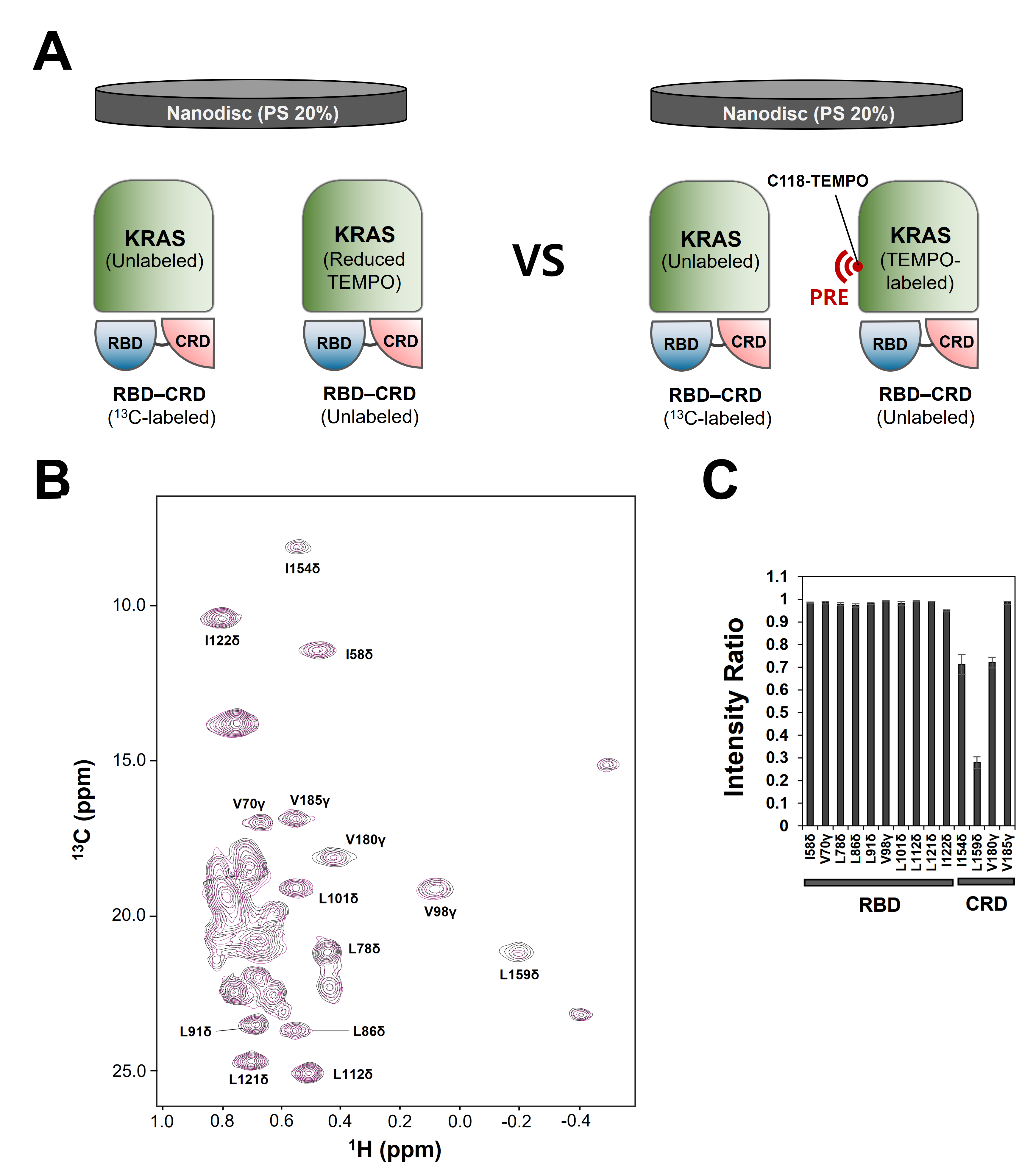


**Figure S7.** PRE-induced reductions in peak intensities for the RBD–CRD in complex with KRAS upon dimerization on the membrane. (A) A schematic of the PRE experimental design with 100 uM [ILV-^13^C methyl]-labeled RBD–CRD complexed with isotopically unlabeled KRAS (KRAS: ^13^C RBD–CRD) and 100 uM KRAS:RBD–CRD tagged with TEMPO at Cys118 of KRAS in the presence of 100 uM lipid leaflets of MSP1E3D1 nanodiscs containing 20% PS. (B) Overlaid ^1^H-^13^C TROSY spectra for KRAS:^13^C RBD–CRD in the presence (blue) and absence (black) of a PRE spin at Cys118 of KRAS in complex with RBD–CRD. (C) Plots of the intensity ratios of the peaks for ILV ^13^C-methyl probes in the spectra in panel (B).


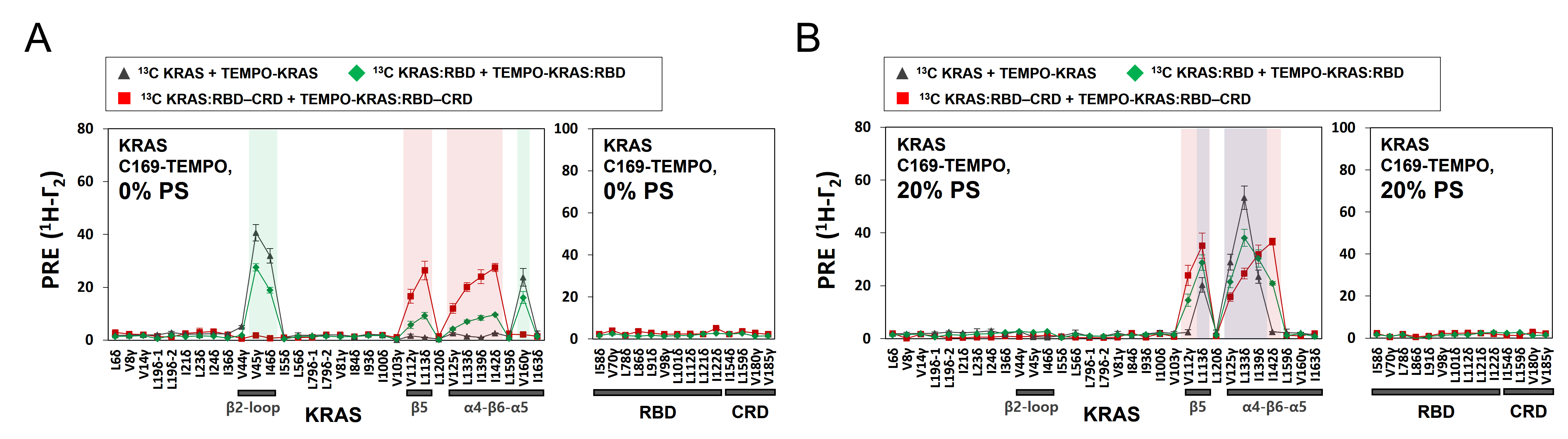


**Figure S8.** Effector-dependent modulation of KRAS dimerization on membranes lacking or containing 20% PS. PRE rates for ILV ^13^C-methyl probes in KRAS, KRAS:RBD, and KRAS:RBD–CRD induced by the addition of the same construct bearing a TEMPO spin label at Cys169 in KRAS in the presence of nanodiscs either containing (A) or lacking (B) PS. Plots obtained for KRAS, KRAS:RBD, and KRAS:RBD–CRD are colored black, green, and red, respectively. The PRE effects for effector-free KRAS dimers on membranes lacking or containing 20% PS and those for the KRAS:RBD–CRD complex on both membranes are indicated by rectangular boxes shaded in green, blue, and red, respectively. Overlay of the PRE effects for free and effector-bound KRAS dimers is shown in the box shaded in violet.

**
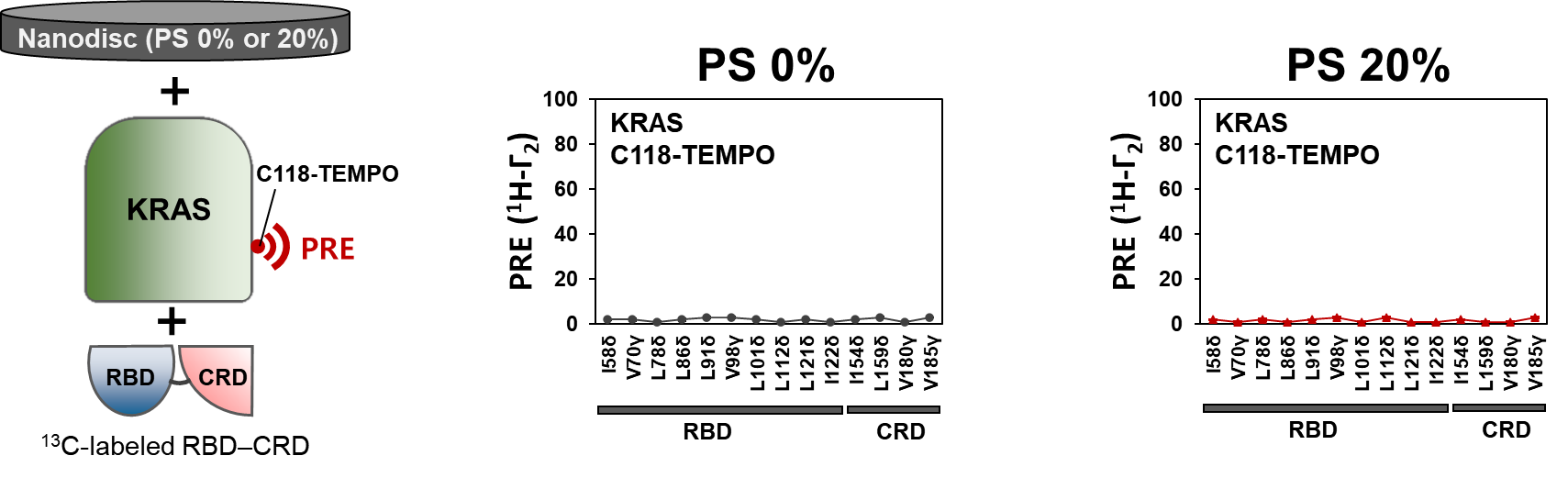
**

**A**

**
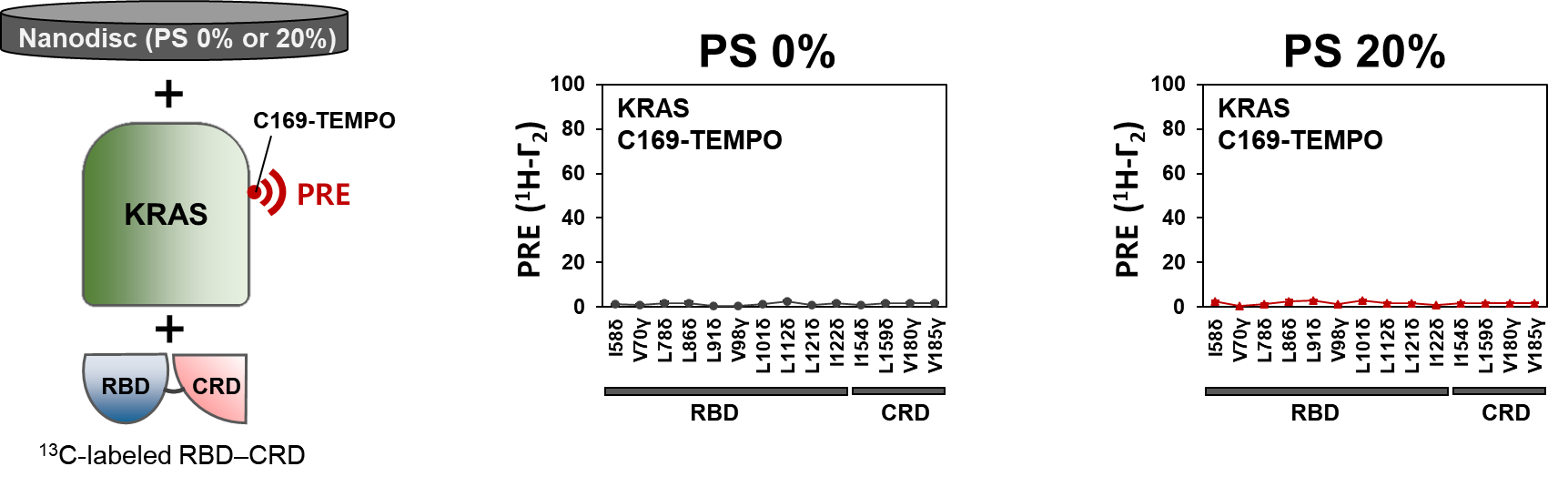
**

**B**

**Figure S9.** No intermolecular PRE effects are induced by the heterodimeric interaction between ^13^C-labelled RBD–CRD and KRAS tagged with TEMPO spin labels at Cys118 (A) or Cys169 (B) at the α4-α5 region (α-interface) in the presence of membranes either containing or lacking 20% PS. PRE rates for ILV ^13^C-methyl probes in the RBD–CRD of RAF1 were measured upon addition of KRAS bearing a TEMPO spin label at Cys118 or Cys169 in the presence of nanodiscs either containing or lacking PS lipid.

**
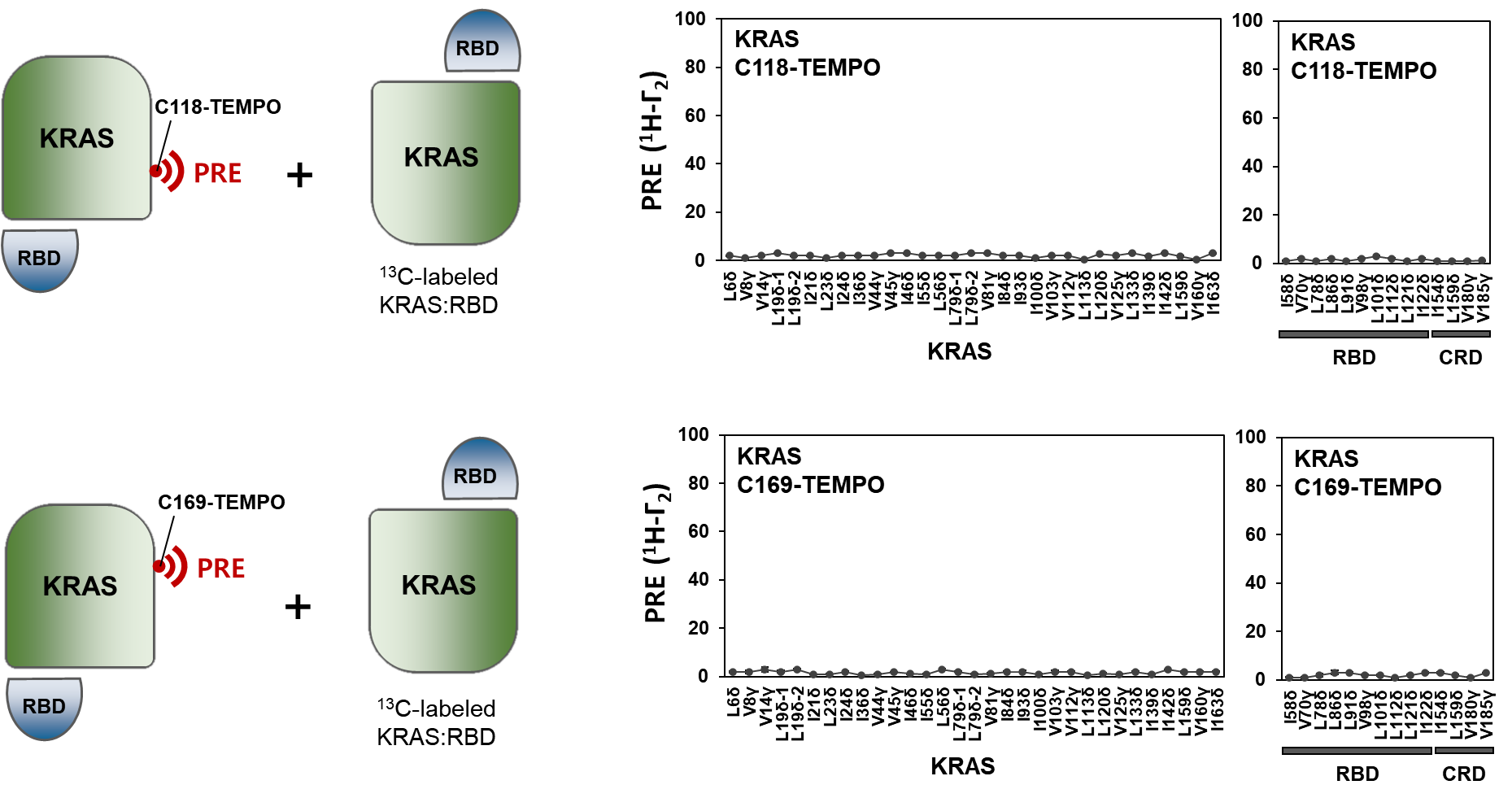
**

**A**

**B**

**
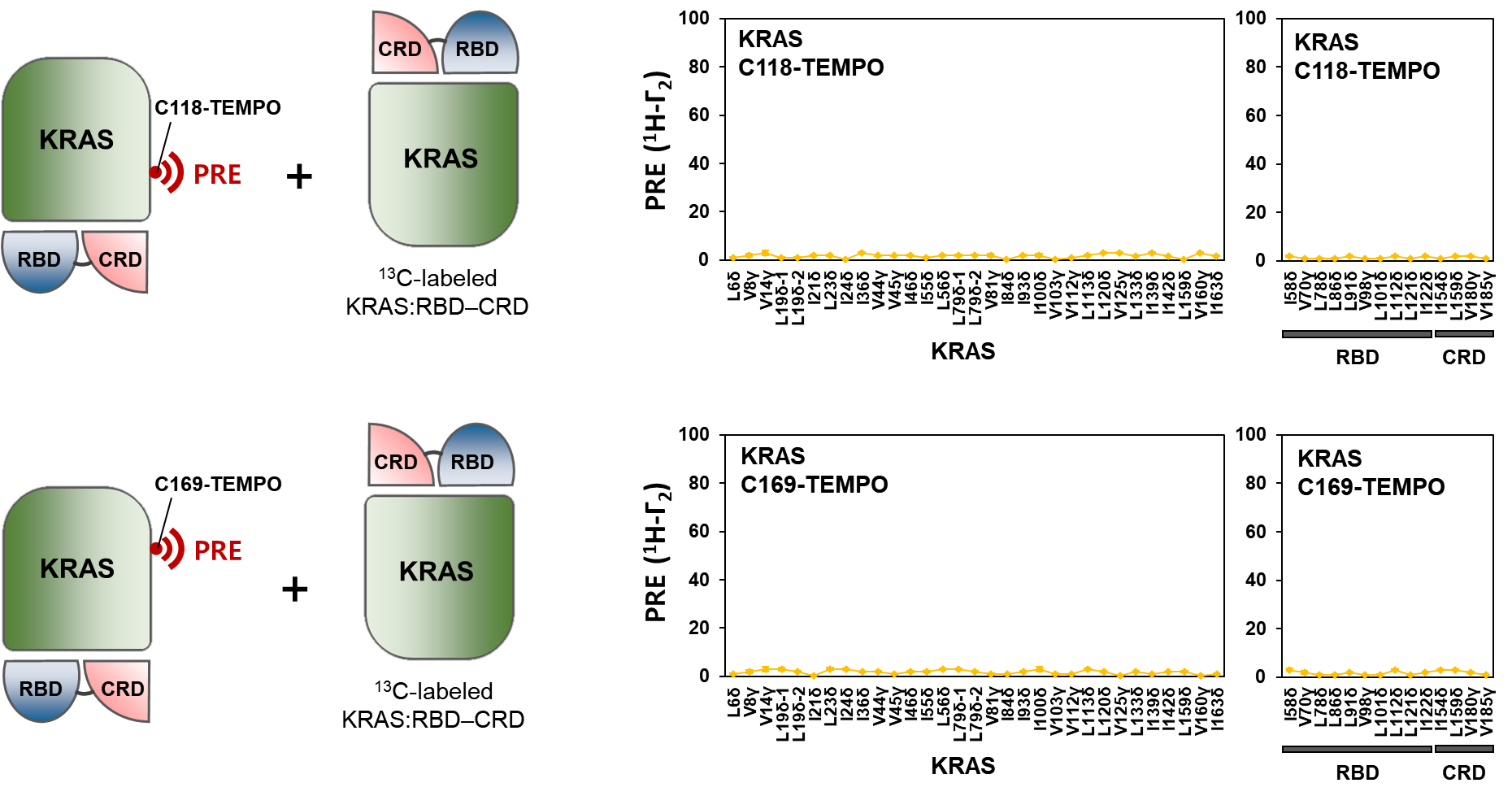
**

**Figure S10.** No intermolecular PRE effects are induced by dimerisation between KRAS molecules in complex with the RBD (A) or RBD–CRD (B) domains in the absence of nanodiscs. PRE rates for ILV ^13^C-methyl probes in the KRAS:RBD–CRD complex were measured upon addition of the KRAS:RBD–CRD complex bearing a TEMPO spin label at Cys118 or Cys169 of KRAS in the absence of nanodiscs.


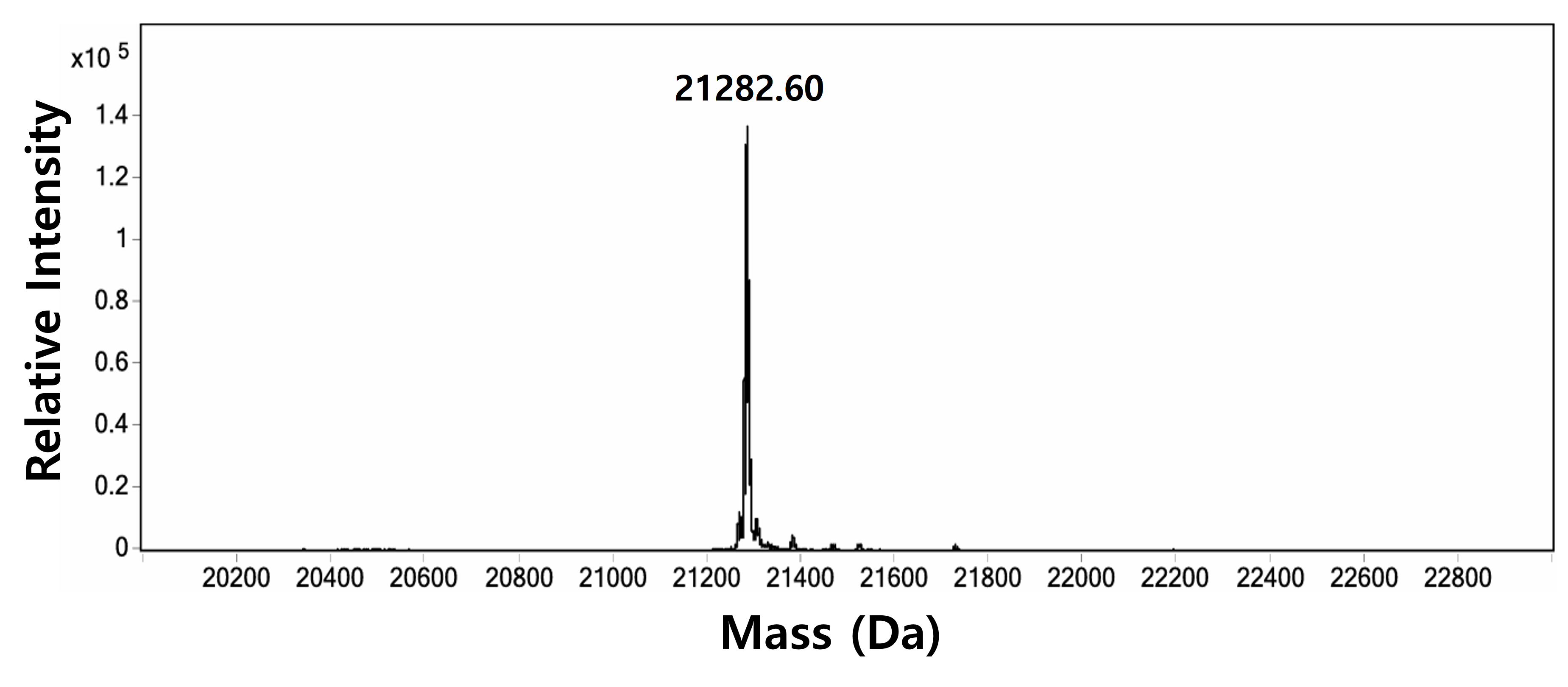


**Figure S11.** Electrospray ionization-mass spectrometry (ESI-MS) detection of fully processed native KRAS purified from Hi5 insect cells showing C-terminal farnesylation and carboxy-methylation with a molecular weight of 21282 Da. The C-terminal farnesylated form of KRAS with a molecular weight of 21268 Da was not detected.


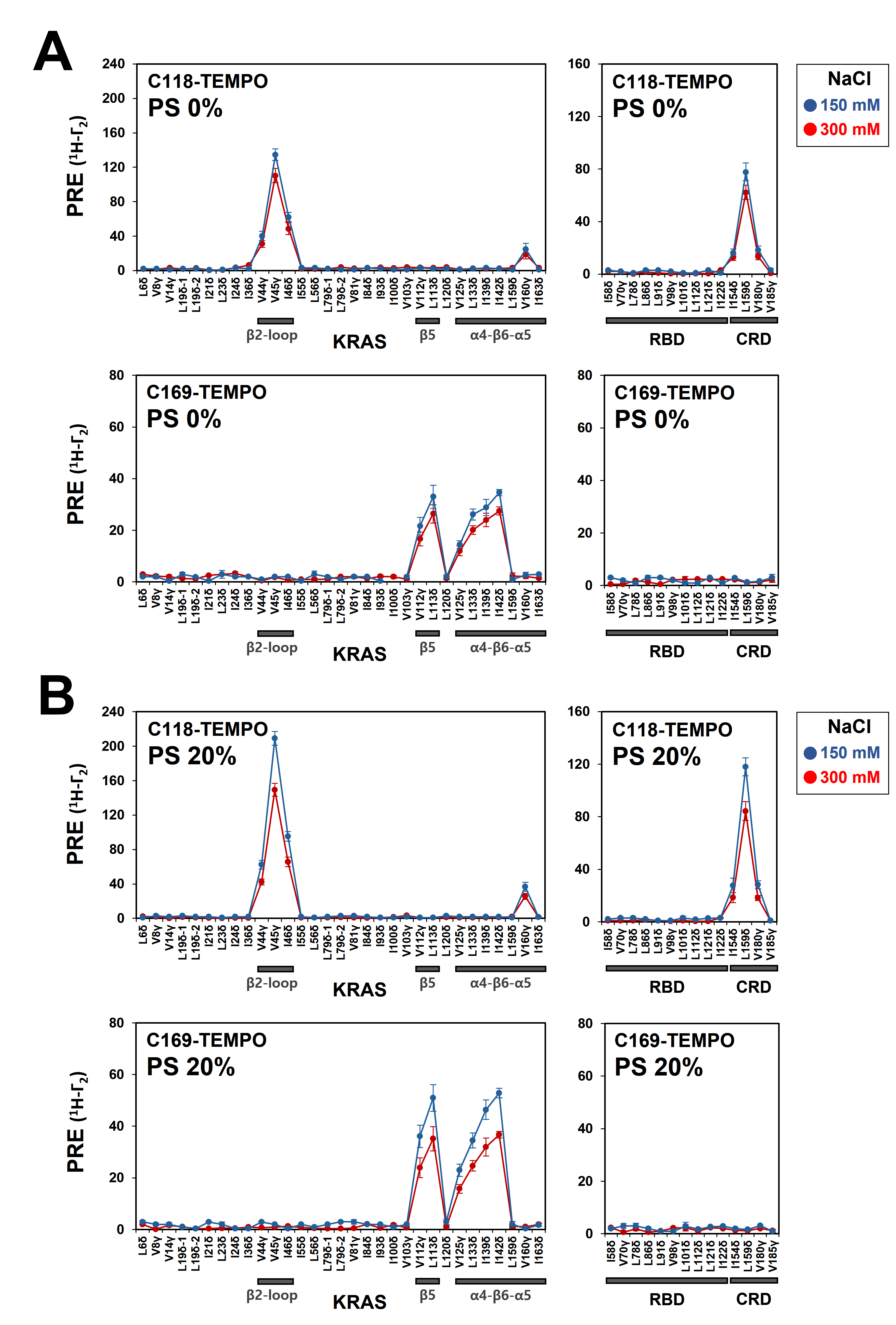


**Figure S12.** The effect of ionic strength on dimerization of the KRAS:RBD–CRD complex on the membrane containing (A) or lacking (B) 20% PS. (A) PRE rates for ILV ^13^C methyl probes of KRAS:RBD–CRD were induced by dimerization with KRAS:RBD–CRD with a TEMPO spin at either Cys118 or Cys169 of KRAS in the presence of 150 mM versus 300 mM NaCl, as indicated.


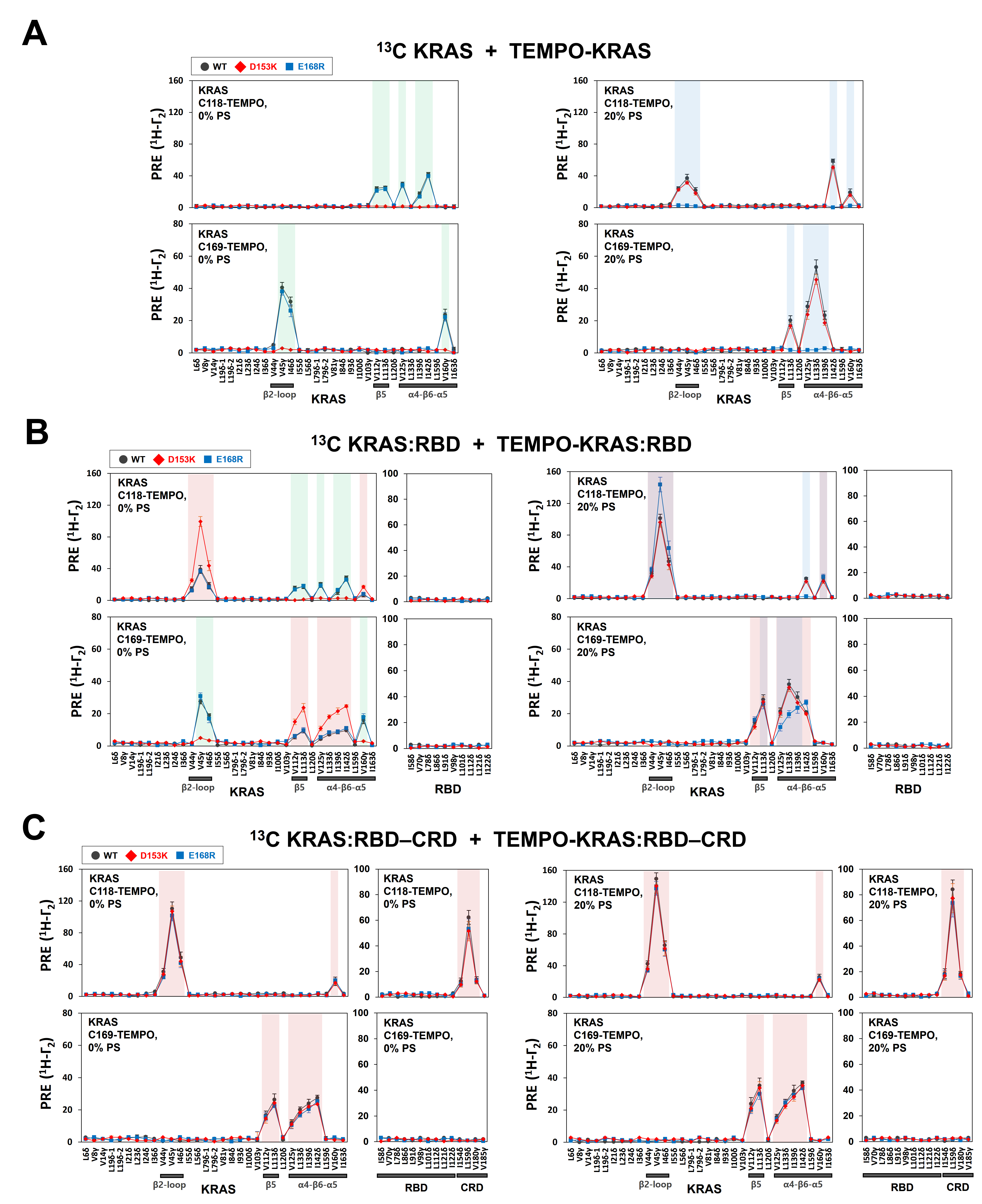


**Figure S13.** Mutagenesis-based selection of a new dimer interface of KRAS in complex with the RBD or RBD–CRD domains of RAF1 on the membrane. PRE rates for ILV 13C-methyl probes in KRAS (A), KRAS:RBD (B) or KRAS:RBD–CRD (C) and their interfacial charge-reversal mutants (D153K and E168R in KRAS) were measured in the presence of the same construct with a TEMPO spin label at either Cys118 or Cys169 and the membrane lacking or containing 20% PS, as indicated. Plots obtained with wild-type KRAS and the D153K and E168R mutants are colored black, red and blue, respectively. The PRE effects for effector-free KRAS dimers on membranes lacking or containing 20% PS and those of the KRAS:RBD–CRD complex on both membranes are indicated by rectangular boxes shaded in green, blue, and red, respectively. Overlay of the PRE effects for free and effector-bound KRAS dimers is shown in the box shaded in violet.


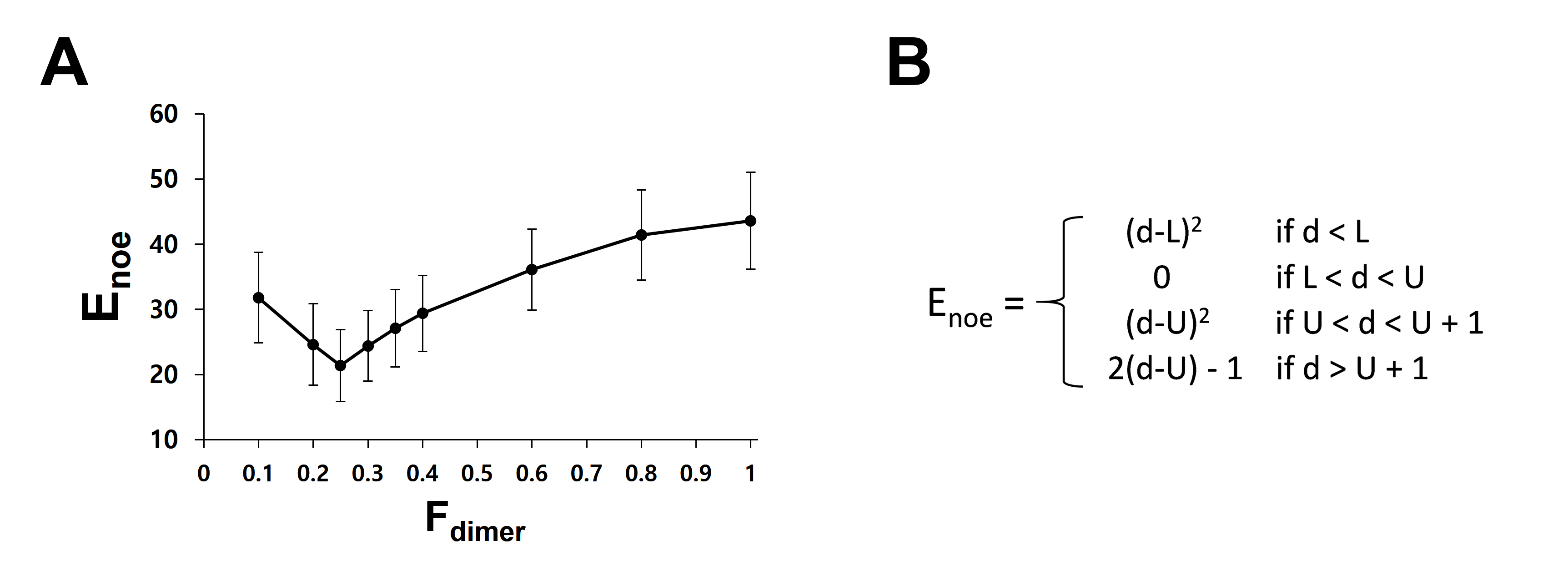


**Figure S14.** Optimization of dimer models the KRAS:RBD–CRD complex using the sets of distance restraints calculated using variable values for the fraction of KRAS that is dimeric (F_dimer_). (A) NOE distance restraint potential (E_noe_) of the 200 lowest Haddock-score structures versus fraction of dimer (F_dimer_). Nine F_dimer_ values, 0.1, 0.2, 0.25, 0.3, 0.35, 0.4, 0.6, 0.8, and 1.0, were used as inputs for the calculation of the corresponding sets of PRE-derived distances in equation 2 in the Methods and Materials section. (B) Conditional formulas to calculate E_noe_. “L” and “U” represent the lower and upper limits of distance restraints, respectively, and “d” indicates the corresponding distance in the final HADDOCK models.

**
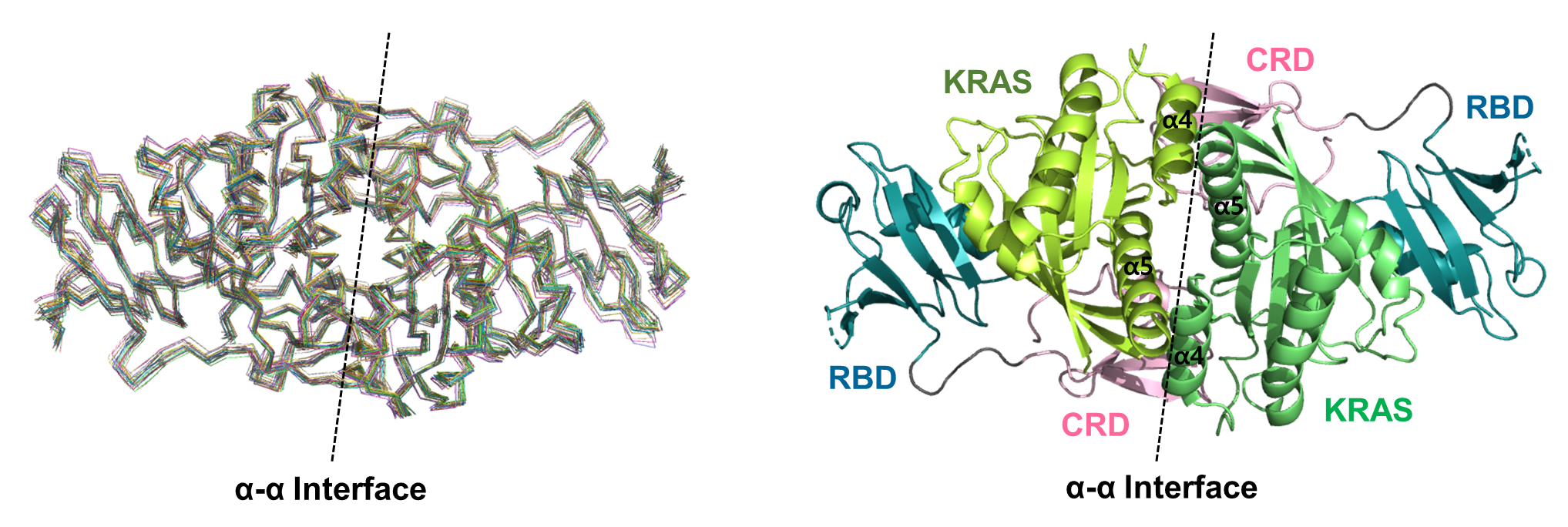
**

**B**

**A**

**Figure S15.** Overlay of the 20 lowest HADDOCK-score models (obtained with the optimal F_dimer_ value of 0.25) (A) and the representative structure (B) of the KRAS dimer in complex with the tandem RBD–CRD domain of RAF1. An average backbone RMSD value for the KRAS GTPase domain (residues 1–172) in complex with the RBD–CRD is 0.94 ± 0.18 Å to the mean structures.


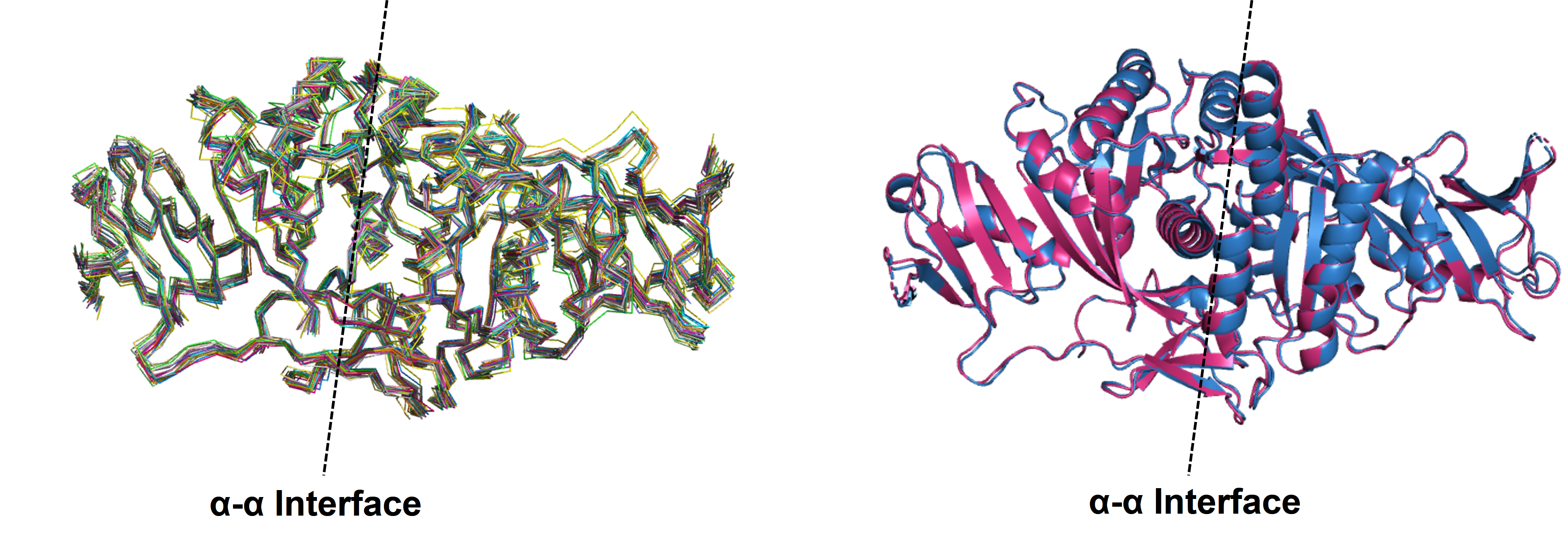


**A**

**B**

**Figure S16.** (A) Overlay of the 20 lowest HADDOCK-score models of the KRAS dimer in complex with the tandem RBD–CRD domain of RAF1 obtained with the F_dimer_ value of 1. An average backbone RMSD value for the KRAS GTPase domain (residues 1–172) in complex with the RBD–CRD is 1.24 ± 0.21 Å to the mean structures. (B) Overlay of the representative structures of the KRAS:RBD–CRD dimers obtained with the F_dimer_ values of 0.25 and 1. A backbone RMSD value for these two structures is 1.13.

**
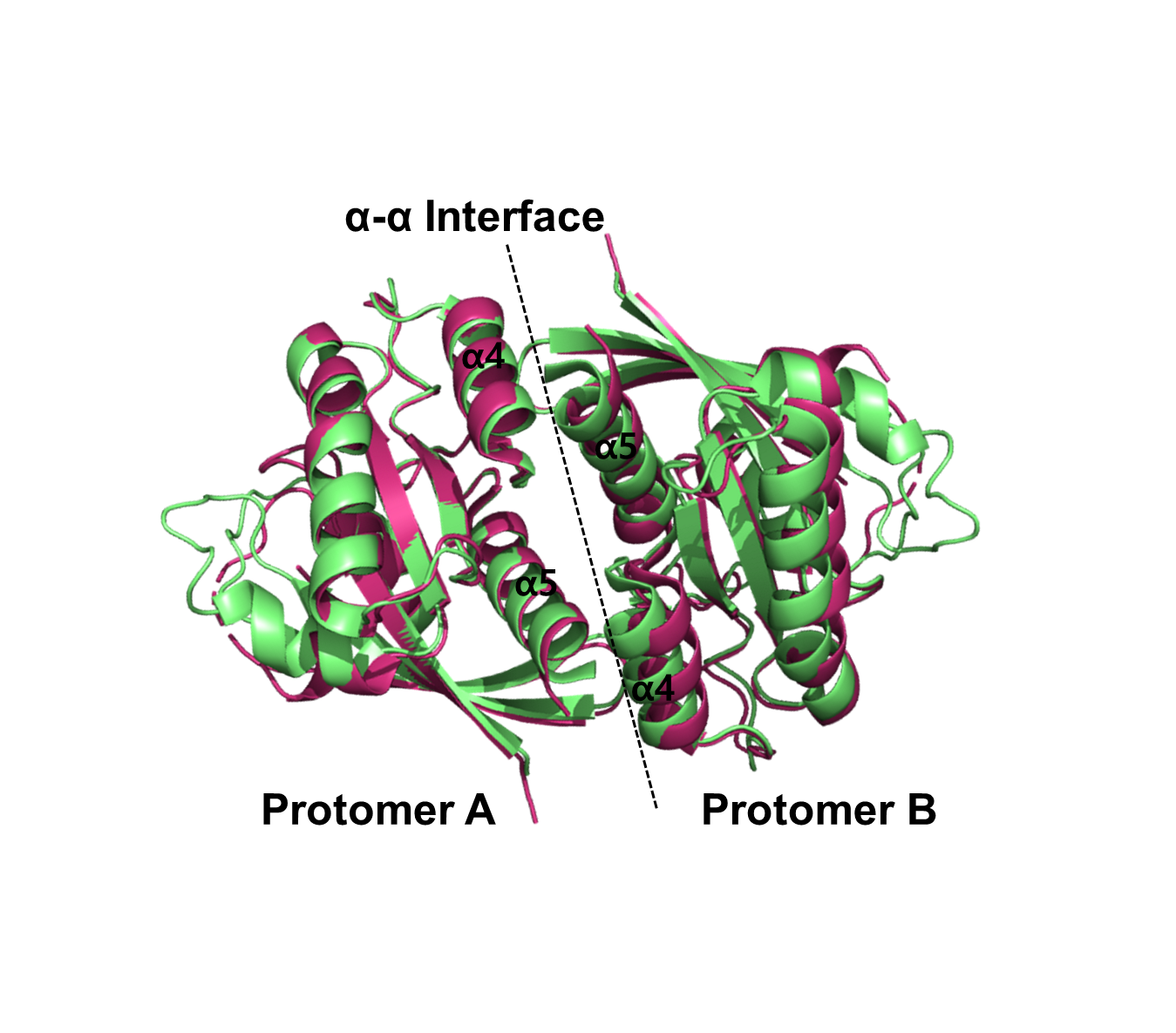
**

**Figure S17.** Comparison between the representative PRE-derived structure of the full-length KRAS dimer in complex with the tandem RBD–CRD domain of RAF1 on the membrane and a crystallographic contact observed for the C-terminally truncated KRAS dimer in the GTP-bound state (PDB ID: 5VQ2). For clarity, only the KRAS GTPase domain (residues 1-168) of the PRE-derived structure is shown. The PRE-derived and crystal structures are coloured green and violet, respectively. One protomer in each KRAS dimer is overlaid to clearly show the different arrangements of the opposing protomers.


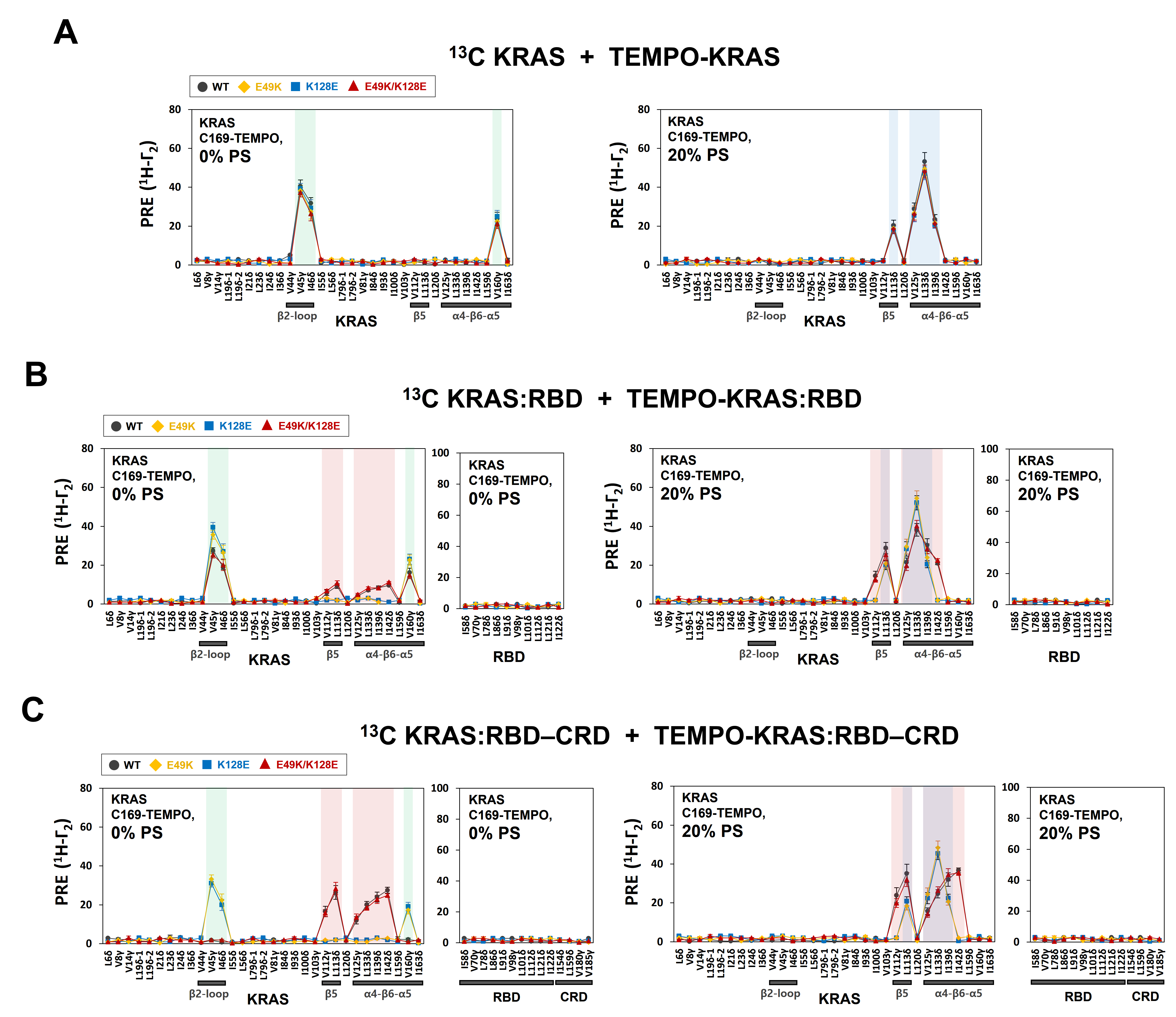


**Figure S18.** Mutagenic validation of the intermolecular interaction specific to the dimer interface of KRAS in complex with the tandem RBD–CRD domain of RAF1 on the membrane. PRE rates for ILV ^13^C-methyl probes in KRAS (A), KRAS:RBD (B) or KRAS:RBD–CRD (C) and their interfacial charge-reversal mutants (E49K, K128E and E49K/K128E in KRAS) were measured in the presence of the same construct with a TEMPO spin label at Cys169 and the membrane lacking or containing 20% PS, as indicated. Plots obtained with wild-type KRAS and the E49K, K128E and E49K/K128E mutants are colored black, yellow, blue, and red, respectively. The PRE effects for effector-free KRAS dimers on membranes lacking or containing 20% PS and those of the KRAS:RBD–CRD complex on both membranes are indicated by rectangular boxes shaded in green, blue, and red, respectively. Overlay of the PRE effects for free and effector-bound KRAS dimers is shown in the box shaded in violet.

**
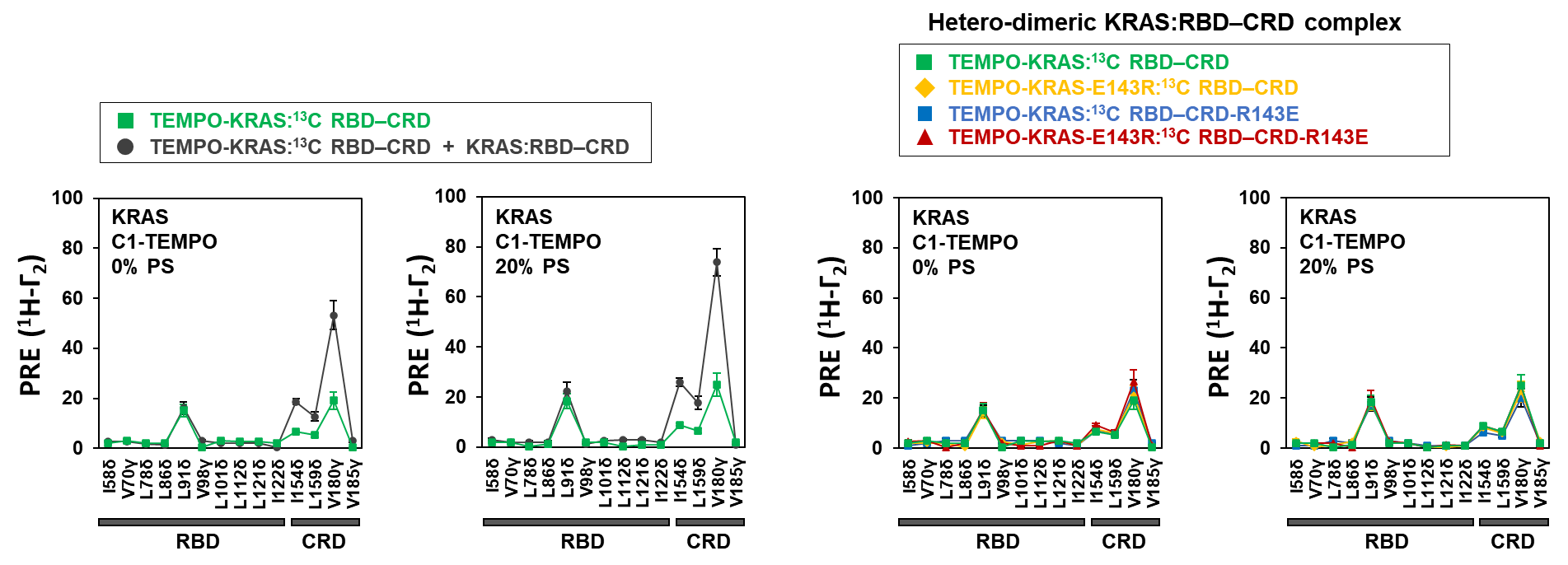
**

**B**

**A**

**Figure S19.** Effects of dimerization of the KRAS:RBD–CRD complex (A) and mutations (R143E in CRD, E143R in KRAS and R143E/E143R) (B) on the KRAS:CRD interaction within the KRAS:RBD–CRD complex. PRE rates for ILV ^13^C-methyl probes in the RBD–CRD in complex with KRAS that is tagged with a TEMPO spin label at Cys1 near the β-sheet effector binding site (β-interface) and those of the mutants were measured in the presence and/or absence of unlabelled KRAS:RBD–CRD complex, as indicated.


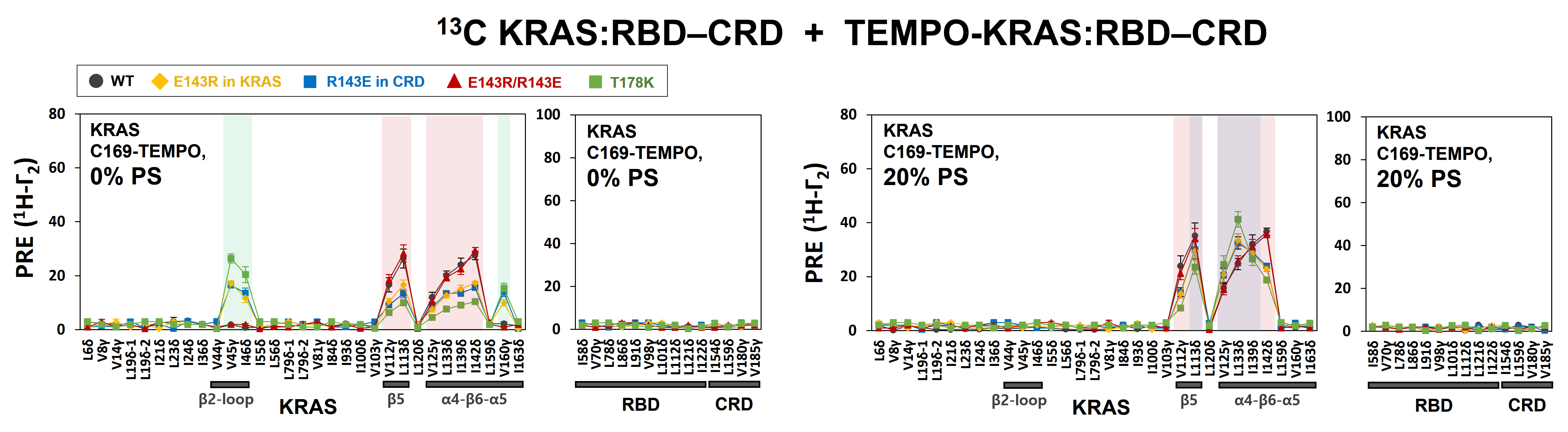


**Figure S20.** Mutagenic validation of key interactions of the CRD for dimerization of the heterodimeric KRAS:RBD–CRD complex. (A) PRE rates for ILV ^13^C-methyl probes in the KRAS:RBD–CRD complex and the interfacial mutants (E143R in KRAS, R143E in CRD, and E143R/R143E and T178K in CRD) in the presence of the same construct with a TEMPO spin label at Cys169 and the membrane lacking or containing 20% PS, as indicated. The PRE effects for effector-free KRAS dimers on membranes lacking or containing 20% PS and those of the KRAS:RBD–CRD complex on both membranes are indicated by rectangular boxes shaded in green, blue, and red, respectively. Overlay of the PRE effects for free and effector-bound KRAS dimers is shown in the box shaded in violet.


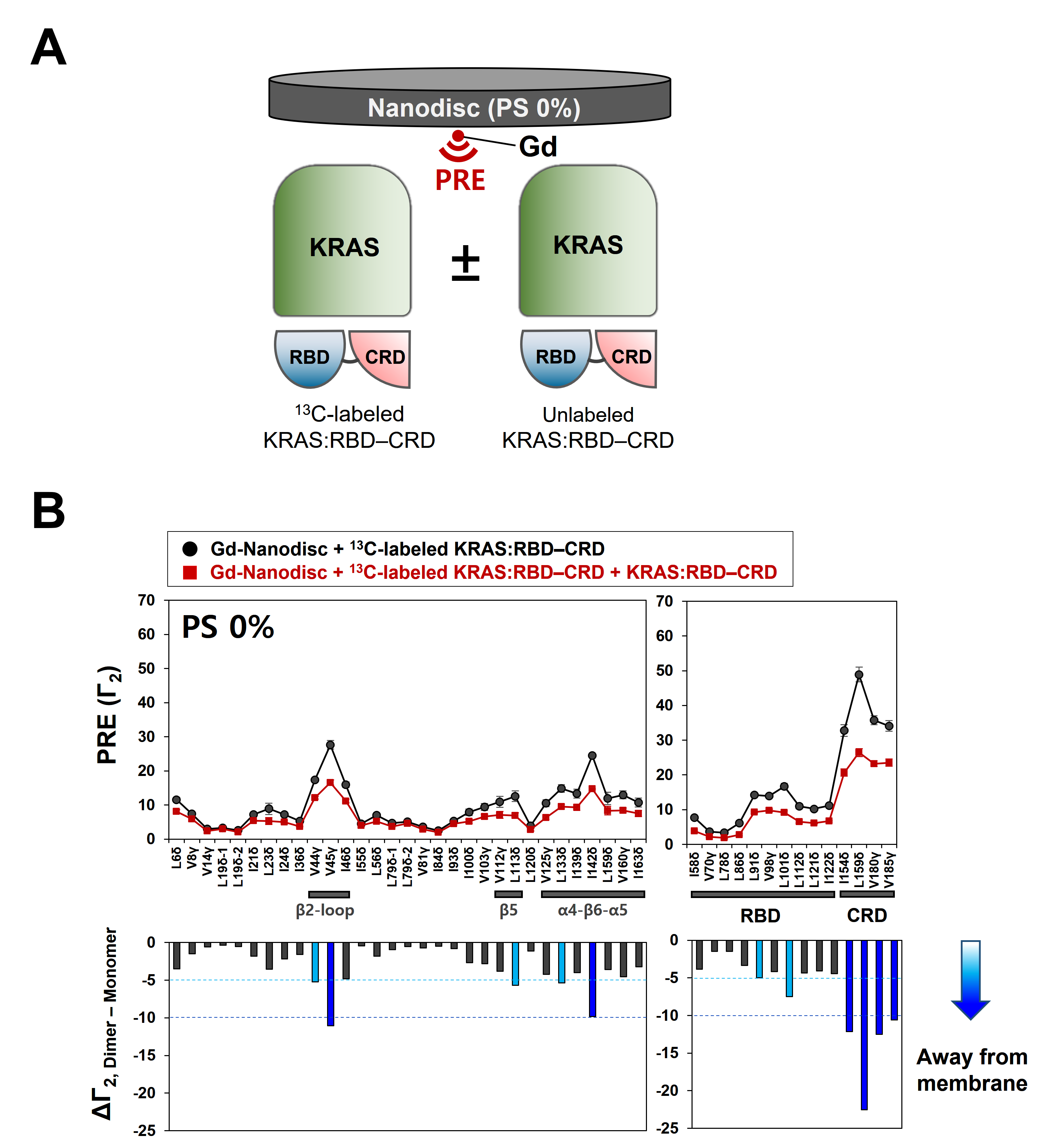


**Figure S21.** Dimerization-induced changes in PRE effects on KRAS:RBD–CRD from spin labels on a neutral membrane surface lacking PS. (A) Illustration of the experimental design with 100 μM [ILV-^13^C methyl]-labeled KRAS:RBD–CRD, 100 μM isotopically unlabeled KRAS:RBD–CRD, and a PS-free, charge-neutral nanodisc (100 μM leaflet) containing a Gd^3+^spin label chelated to the headgroups of PE-DTPA. (B) ^1^H-Γ_2_ PRE rates of ILV ^13^C-methyl probes in KRAS:RBD–CRD induced by Gd^3+^-associated membranes in the presence (red) and absence (black) of an equal amount of unlabeled KRAS:RBD–CRD, and changes in ^1^H-Γ_2_ rates (Δ^1^H-Γ_2_,_dimer-monomer_) upon addition of KRAS:RBD–CRD (lower panel). Negative values of Δ^1^H-Γ_2_,_dimer-monomer_ represent loss of the membrane PRE effect upon dimerization. Probes with PRE changes are categorized according to the threshold values of Δ^1^H-Γ_2_,_dimer-monomer_: < -5 s^-1^ (cyan) and < -10 s^-1^ (blue).


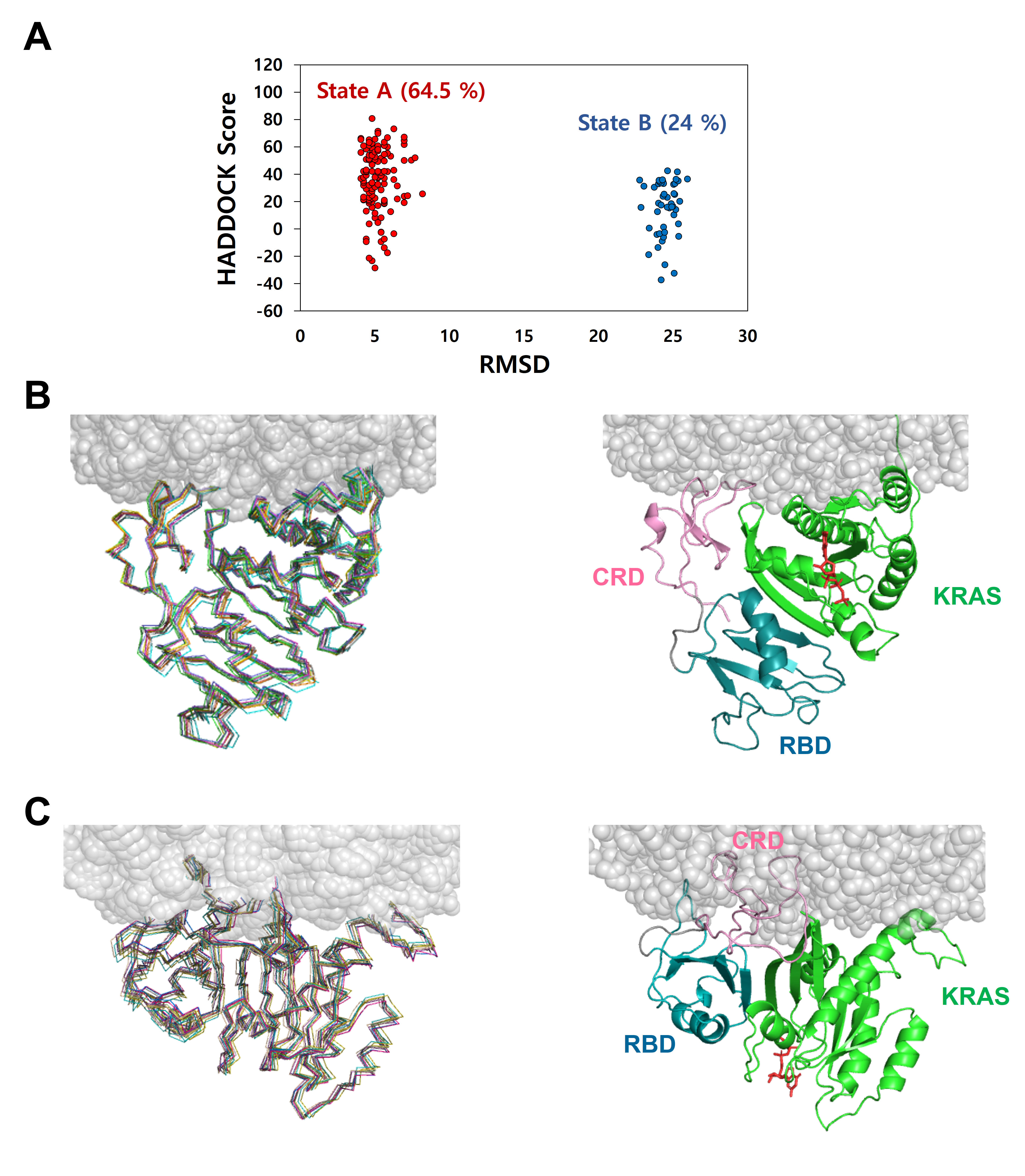
**Figure S22.** PRE-driven structural models of the monomeric KRAS:RBD–CRD complex on anionic membranes containing 20% phosphatidylserine (PS) lipids. (A) Cluster analysis of the 200 lowest HADDOCK-score structures of the monomeric KRAS:RBD–CRD complex on the membrane. HADDOCK scores of the monomeric KRAS:RBD–CRD complex in state A (major) and state B (minor) versus root-mean-square deviation (RMSD) values to the mean structure of state A are plotted. Overlay of the 10 lowest HADDOCK-score structures (left) and the representative structure (right) of the KRAS:RBD–CRD monomer on the membrane in state A (B) and state B (C). Average backbone RMSD value for the KRAS GTPase domain (residues 1–172) in complex with the RBD–CRD is 1.45 ± 0.25 Å for state A and 1.82 ± 0.28 Å for state B to the mean structures.

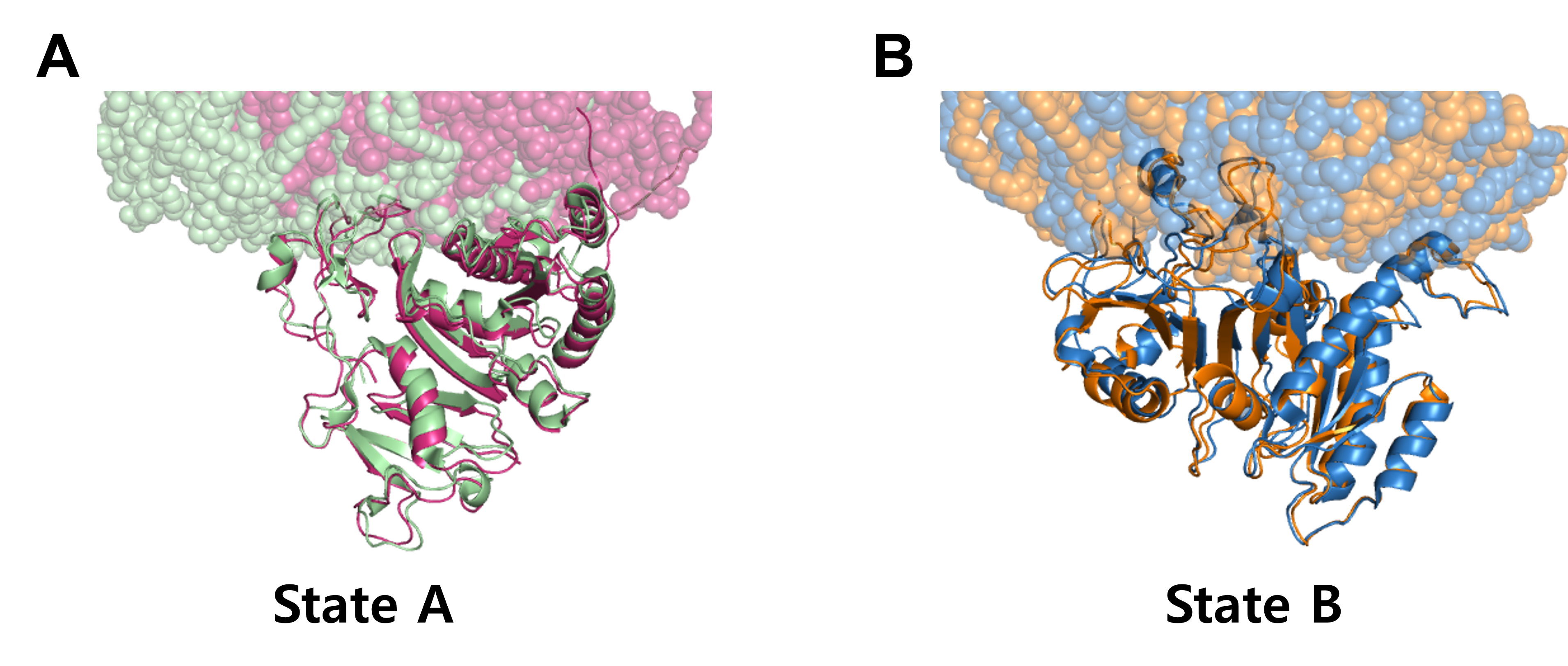


**Figure S23.** Comparison between the representative PRE-derived structures of the KRAS:RBD–CRD monomer on the membrane in state A (A) and state B (B) and the structures (PDB IDs: 6PTS for state A and 6PTW for state B) previously reported using the same approach. The structures in state A and state B obtained in this study are colored green and orange, respectively.


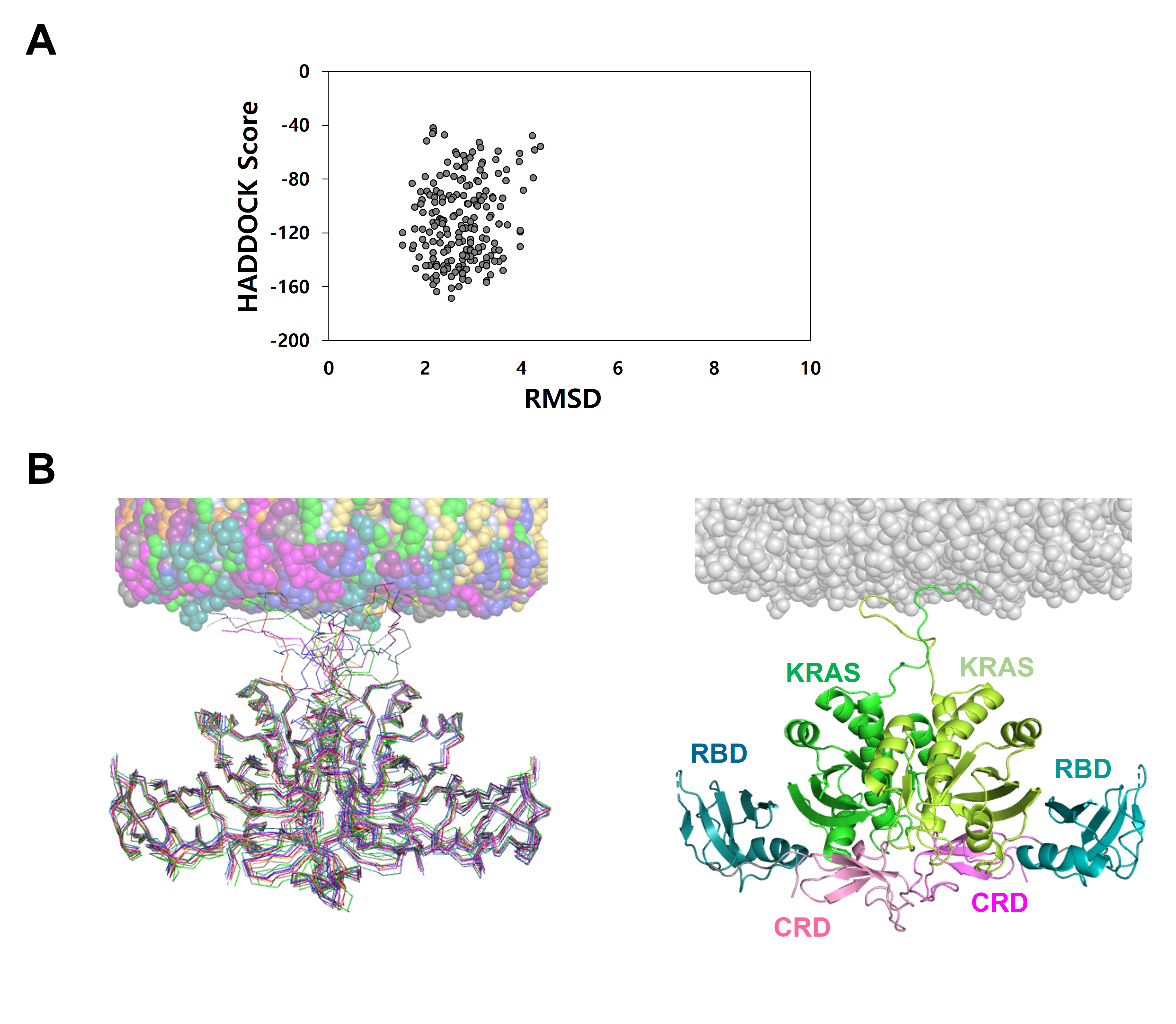


**Figure S24.** PRE-driven structural models of the dimeric KRAS:RBD–CRD complex on anionic membranes containing 20% phosphatidylserine (PS) lipids. (A) HADDOCK scores of the KRAS:RBD–CRD dimer versus root-mean-square deviation (RMSD) values to the mean structure. (B) Overlay of the 10 lowest HADDOCK-score structures of the dimeric KRAS:RBD–CRD complex on anionic membranes containing 20% phosphatidylserine (PS) lipids. An average backbone RMSD value for the KRAS GTPase domain (residues 1–172) in complex with the RBD–CRD is 1.19 ± 0.23 Å to the mean structure.

**3. Supplementary Tables**

**Table S1.** Statistics for the 20 lowest HADDOCK-score structures of the KRAS dimer in complex with the tandem RBD–CRD domain of RAF1.

| **PRE-derived Distance Restraints** | |  |
| --- | --- | --- |
| Number of TEMPO-PRE restraints | 26 | |
| **Energy Statistics** |  | |
| HADDOCK score (a.u.) | -148 ± 14 | |
| Van der Waals energy (kcal/mol) | -153 ± 10 | |
| Electrostatic energy (kcal/mol) | -115 ± 11 | |
| Desolvation energy (kcal/mol) | 42 ± 8 | |
| **Total buried surface area (Å^2^)** | 3416 ± 51 | |

**Table S2**. Violation analysis of PRE-derived restraints of the KRAS:RBD–CRD complex versus the representative PRE-derived structure of the KRAS dimer in complex with the RBD–CRD domain of RAF1.

| **The Structure of the KRAS dimer in complex with the RBD–CRD** | | | | | | | | | **PRE-Derived Distance (Å)** | **Result^#^** |
| --- | --- | --- | --- | --- | --- | --- | --- | --- | --- | --- |
| **Chain** | **Residue Number** | **Atom Name** | **Chain** | **Residue Number** | **Atom Name** | **Distance**  **(Å)** | ***d_eff_**** | **Average Distance (Å)** |  |  |
| KRAS-A | 118 | SG | KRAS-B | 44 | CG1 | 16.0 | 15.0 | 14.9 | 15.6 ± 3.0 | S |
| KRAS-A | 118 | SG | KRAS-B | 44 | CG2 | 18.2 |  |  |  |  |
| KRAS-B | 118 | SG | KRAS-A | 44 | CG1 | 15.8 | 14.9 |  |  |  |
| KRAS-B | 118 | SG | KRAS-A | 44 | CG2 | 18.1 |  |  |  |  |
| KRAS-A | 118 | SG | KRAS-B | 45 | CG1 | 10.9 | 10.4 | 10.5 | 12.4 ± 3.0 | S |
| KRAS-A | 118 | SG | KRAS-B | 45 | CG2 | 13.1 |  |  |  |  |
| KRAS-B | 118 | SG | KRAS-A | 45 | CG1 | 11.2 | 10.7 |  |  |  |
| KRAS-B | 118 | SG | KRAS-A | 45 | CG2 | 13.5 |  |  |  |  |
| KRAS-A | 118 | SG | KRAS-B | 46 | CD1 | 14.8 |  | 14.7 | 14.2 ± 3.0 | S |
| KRAS-B | 118 | SG | KRAS-A | 46 | CD1 | 14.6 |  |  |  |  |
| KRAS-A | 118 | SG | KRAS-B | 160 | CG1 | 17.4 | 16.1 | 16.4 | 16.6 ± 3.0 | S |
| KRAS-A | 118 | SG | KRAS-B | 160 | CG2 | 18.8 |  |  |  |  |
| KRAS-B | 118 | SG | KRAS-A | 160 | CG1 | 18.1 | 16.7 |  |  |  |
| KRAS-B | 118 | SG | KRAS-A | 160 | CG2 | 19.5 |  |  |  |  |
| KRAS-A | 118 | SG | CRD-B | 154 | CD1 | 18.3 |  | 18.2 | 18.3 ± 3.0 | S |
| KRAS-B | 118 | SG | CRD-A | 154 | CD1 | 18.1 |  |  |  |  |
| KRAS-A | 118 | SG | CRD-B | 159 | CD1 | 12.2 | 11.6 | 11.3 | 13.6 ± 3.0 | S |
| KRAS-A | 118 | SG | CRD-B | 159 | CD2 | 14.3 |  |  |  |  |
| KRAS-B | 118 | SG | CRD-A | 159 | CD1 | 11.5 | 11.0 |  |  |  |
| KRAS-B | 118 | SG | CRD-A | 159 | CD2 | 13.9 |  |  |  |  |
| KRAS-A | 118 | SG | CRD-B | 180 | CD1 | 20.1 | 16.8 | 16.6 | 17.6 ± 3.0 | S |
| KRAS-A | 118 | SG | CRD-B | 180 | CD2 | 18.1 |  |  |  |  |
| KRAS-B | 118 | SG | CRD-A | 180 | CD1 | 19.5 | 16.4 |  |  |  |
| KRAS-B | 118 | SG | CRD-A | 180 | CD2 | 17.6 |  |  |  |  |
| KRAS-A | 169 | CG | KRAS-B | 112 | CG1 | 18.0 | 16.5 | 16.6 | 17.1 ± 3.0 | S |
| KRAS-A | 169 | CG | KRAS-B | 112 | CG2 | 19.1 |  |  |  |  |
| KRAS-B | 169 | CG | KRAS-A | 112 | CG1 | 18.3 | 16.7 |  |  |  |
| KRAS-B | 169 | CG | KRAS-A | 112 | CG2 | 19.4 |  |  |  |  |
| KRAS-A | 169 | CG | KRAS-B | 113 | CD1 | 18.5 | 15.1 | 14.9 | 15.8 ± 3.0 | S |
| KRAS-A | 169 | CG | KRAS-B | 113 | CD2 | 16.0 |  |  |  |  |
| KRAS-B | 169 | CG | KRAS-A | 113 | CD1 | 18.3 | 14.7 |  |  |  |
| KRAS-B | 169 | CG | KRAS-A | 113 | CD2 | 15.5 |  |  |  |  |
| KRAS-A | 169 | CG | KRAS-B | 125 | CG1 | 18.3 | 17.1 | 16.7 | 18.0 ± 3.0 | S |
| KRAS-A | 169 | CG | KRAS-B | 125 | CG2 | 20.8 |  |  |  |  |
| KRAS-B | 169 | CG | KRAS-A | 125 | CG1 | 17.5 | 16.3 |  |  |  |
| KRAS-B | 169 | CG | KRAS-A | 125 | CG2 | 19.4 |  |  |  |  |
| KRAS-A | 169 | CG | KRAS-B | 133 | CD1 | 17.9 | 16.0 | 15.9 | 16.5 ± 3.0 | S |
| KRAS-A | 169 | CG | KRAS-B | 133 | CD2 | 18.1 |  |  |  |  |
| KRAS-B | 169 | CG | KRAS-A | 133 | CD1 | 17.6 | 15.8 |  |  |  |
| KRAS-B | 169 | CG | KRAS-A | 133 | CD2 | 17.8 |  |  |  |  |
| KRAS-A | 169 | CG | KRAS-B | 139 | CD1 | 16.6 |  | 16.5 | 16.0 ± 3.0 | S |
| KRAS-B | 169 | CG | KRAS-A | 139 | CD1 | 16.4 |  |  |  |  |
| KRAS-A | 169 | CG | KRAS-B | 142 | CD1 | 15.4 |  | 15.7 | 15.7 ± 3.0 | S |
| KRAS-B | 169 | CG | KRAS-A | 142 | CD1 | 15.9 |  |  |  |  |
| KRAS-A | 1 | CG | CRD-A | 91 | CD1 | 20.2 | 17.6 | 17.3 | 17.0 ± 3.0 | S |
| KRAS-A | 1 | CG | CRD-A | 91 | CD2 | 19.4 |  |  |  |  |
| KRAS-B | 1 | CG | CRD-B | 91 | CD1 | 19.5 | 17.0 |  |  |  |
| KRAS-B | 1 | CG | CRD-B | 91 | CD2 | 18.8 |  |  |  |  |
| KRAS-A | 1 | CG | CRD-A | 154 | CD1 | 17.6 |  | 17.5 | 16.8 ± 3.0 | S |
| KRAS-B | 1 | CG | CRD-B | 154 | CD1 | 17.4 |  |  |  |  |
| KRAS-A | 1 | CG | CRD-A | 159 | CD1 | 21.2 | 18.9 | 18.5 | 18.1 ± 3.0 | S |
| KRAS-A | 1 | CG | CRD-A | 159 | CD2 | 21.2 |  |  |  |  |
| KRAS-B | 1 | CG | CRD-B | 159 | CD1 | 20.4 | 18.1 |  |  |  |
| KRAS-B | 1 | CG | CRD-B | 159 | CD2 | 20.3 |  |  |  |  |
| KRAS-A | 1 | CG | CRD-A | 180 | CG1 | 15.4 | 13.9 | 14.0 | 12.2 ± 3.0 | S |
| KRAS-A | 1 | CG | CRD-A | 180 | CG2 | 15.8 |  |  |  |  |
| KRAS-B | 1 | CG | CRD-B | 180 | CG1 | 15.6 | 14.1 |  |  |  |
| KRAS-B | 1 | CG | CRD-B | 180 | CG2 | 16.1 |  |  |  |  |

^*^An effective distance (*d_eff_*) for ambiguous interactions was calculated with the following formula: $d_{eff}=\sum_{i=1}^{N_{atoms}} {(\frac{1}{d_{i}^{6}})}^{-\frac{1}{6}}$ where *N_atoms_* indicates all atoms that are involved in ambiguous interactions.

^#^ “S” represents satisfaction and violation of experimental PRE distance restraints by the PRE-derived structure of the KRAS dimer in complex with the RBD–CRD.

**Table S3.** Intermolecular interactions within KRAS:KRAS or KRAS:CRD interfaces to mediate dimerisation of the KRAS:RBD–CRD complex in the 10 lowest HADDOCK-score structures of the hetero-tetrameric KRAS:RBD–CRD complex.

| **Model 1** | | | | | | | | |
| --- | --- | --- | --- | --- | --- | --- | --- | --- |
| **Chain** | **Residue Number** | **Residue Name** | **Atom Name** | **Chain** | **Residue Number** | **Residue Name** | **Atom Name** | **Distance^*^ (Å)** |
| KRAS-A | 47 | ASP | OD1 | KRAS-B | 143 | GLU | HN | 1.8 |
| KRAS-A | 49 | GLU | OE1 | KRAS-B | 128 | LYS | HZ1 | 1.6 |
| KRAS-A | 49 | GLU | OE1 | KRAS-B | 131 | GLN | HE22 | 1.9 |
| KRAS-A | 128 | LYS | HZ1 | KRAS-B | 169 | LYS | O | 1.7 |
| KRAS-A | 128 | LYS | HZ2 | KRAS-B | 2 | THR | OG1 | 2.2 |
| KRAS-A | 128 | LYS | HZ3 | KRAS-B | 49 | GLU | OE1 | 1.6 |
| KRAS-A | 131 | GLN | HE22 | KRAS-B | 49 | GLU | OE1 | 1.8 |
| KRAS-A | 135 | ARG | HH12 | KRAS-B | 164 | ARG | O | 2.1 |
| KRAS-A | 143 | GLU | HN | KRAS-B | 47 | ASP | OD1 | 1.9 |
| KRAS-A | 148 | THR | HG1 | CRD-B | 161 | ASN | OD1 | 2.4 |
| KRAS-A | 150 | GLN | HE21 | CRD-B | 174 | GLU | OE2 | 2.4 |
| KRAS-A | 154 | ASP | OD1 | KRAS-B | 161 | ARG | HH12 | 1.8 |
| KRAS-A | 154 | ASP | OD2 | KRAS-B | 161 | ARG | HH12 | 2.3 |
| KRAS-A | 154 | ASP | OD2 | KRAS-B | 161 | ARG | HH22 | 1.7 |
| KRAS-A | 154 | ASP | OD2 | CRD-B | 175 | HIS | HE2 | 1.7 |
| KRAS-A | 161 | ARG | HH11 | KRAS-B | 154 | ASP | OD1 | 2.0 |
| KRAS-A | 161 | ARG | HH11 | KRAS-B | 154 | ASP | OD2 | 2.1 |
| KRAS-A | 161 | ARG | HH21 | KRAS-B | 154 | ASP | OD2 | 1.7 |
| KRAS-A | 164 | ARG | O | KRAS-B | 135 | ARG | HH11 | 1.8 |
| KRAS-A | 169 | LYS | O | KRAS-B | 128 | LYS | HZ2 | 1.8 |
| CRD-A | 143 | ARG | HH11 | KRAS-B | 143 | GLU | OE1 | 1.6 |
| CRD-A | 175 | HIS | HN | KRAS-B | 150 | GLN | OE1 | 2.2 |
| CRD-A | 175 | HIS | HE2 | KRAS-B | 154 | ASP | OD2 | 1.7 |
| **Model 2** | | | | | | | | |
| **Chain** | **Residue Number** | **Residue Name** | **Atom Name** | **Chain** | **Residue Number** | **Residue Name** | **Atom Name** | **Distance^*^ (Å)** |
| KRAS-A | 47 | ASP | OD1 | KRAS-B | 143 | GLU | HN | 1.8 |
| KRAS-A | 47 | ASP | O | KRAS-B | 127 | THR | HG1 | 2.5 |
| KRAS-A | 49 | GLU | OE1 | KRAS-B | 128 | LYS | HZ1 | 1.7 |
| KRAS-A | 49 | GLU | OE2 | KRAS-B | 128 | LYS | HZ1 | 2.4 |
| KRAS-A | 49 | GLU | OE2 | KRAS-B | 131 | GLN | HE22 | 1.7 |
| KRAS-A | 127 | THR | HG1 | KRAS-B | 47 | ASP | O | 2.2 |
| KRAS-A | 128 | LYS | HZ1 | KRAS-B | 169 | LYS | O | 1.8 |
| KRAS-A | 128 | LYS | HZ2 | KRAS-B | 2 | THR | OG1 | 1.9 |
| KRAS-A | 128 | LYS | HZ3 | KRAS-B | 49 | GLU | OE1 | 1.6 |
| KRAS-A | 131 | GLN | HE22 | KRAS-B | 49 | GLU | OE1 | 1.8 |
| KRAS-A | 135 | ARG | HH12 | KRAS-B | 164 | ARG | O | 2.0 |
| KRAS-A | 135 | ARG | HH12 | KRAS-B | 167 | LYS | O | 2.3 |
| KRAS-A | 143 | GLU | HN | KRAS-B | 47 | ASP | OD1 | 1.8 |
| KRAS-A | 148 | THR | HG1 | CRD-B | 161 | ASN | OD1 | 2.1 |
| KRAS-A | 154 | ASP | OD1 | KRAS-B | 161 | ARG | HH12 | 2.0 |
| KRAS-A | 154 | ASP | OD2 | KRAS-B | 161 | ARG | HH12 | 1.9 |
| KRAS-A | 154 | ASP | OD2 | KRAS-B | 161 | ARG | HH22 | 1.8 |
| KRAS-A | 154 | ASP | OD2 | CRD-B | 175 | HIS | HE2 | 1.8 |
| KRAS-A | 161 | ARG | HH11 | KRAS-B | 154 | ASP | OD1 | 1.7 |
| KRAS-A | 161 | ARG | HH11 | KRAS-B | 154 | ASP | OD2 | 2.3 |
| KRAS-A | 161 | ARG | HH21 | KRAS-B | 154 | ASP | OD2 | 1.6 |
| KRAS-A | 169 | LYS | O | KRAS-B | 128 | LYS | HZ3 | 1.7 |
| CRD-A | 143 | ARG | HH11 | KRAS-B | 143 | GLU | OE1 | 2.1 |
| CRD-A | 175 | HIS | HE2 | KRAS-B | 154 | ASP | OD2 | 1.9 |
| **Model 3** | | | | | | | | |
| **Chain** | **Residue Number** | **Residue Name** | **Atom Name** | **Chain** | **Residue Number** | **Residue Name** | **Atom Name** | **Distance^*^ (Å)** |
| KRAS-A | 2 | THR | OG1 | KRAS-B | 128 | LYS | HZ2 | 2.0 |
| KRAS-A | 47 | ASP | OD1 | KRAS-B | 143 | GLU | HN | 1.7 |
| KRAS-A | 47 | ASP | O | KRAS-B | 127 | THR | HG1 | 2.3 |
| KRAS-A | 49 | GLU | OE1 | KRAS-B | 128 | LYS | HZ3 | 1.6 |
| KRAS-A | 49 | GLU | OE1 | KRAS-B | 131 | GLN | HE22 | 1.9 |
| KRAS-A | 127 | THR | HG1 | KRAS-B | 47 | ASP | O | 2.4 |
| KRAS-A | 128 | LYS | HZ1 | KRAS-B | 2 | THR | OG1 | 1.9 |
| KRAS-A | 128 | LYS | HZ3 | KRAS-B | 49 | GLU | OE1 | 1.6 |
| KRAS-A | 131 | GLN | HE22 | KRAS-B | 49 | GLU | OE1 | 2.1 |
| KRAS-A | 135 | ARG | HH12 | KRAS-B | 164 | ARG | O | 2.2 |
| KRAS-A | 143 | GLU | HN | KRAS-B | 47 | ASP | OD1 | 1.8 |
| KRAS-A | 143 | GLU | OE1 | CRD-B | 143 | ARG | HH11 | 2.4 |
| KRAS-A | 154 | ASP | OD1 | KRAS-B | 161 | ARG | HH12 | 2.3 |
| KRAS-A | 154 | ASP | OD2 | KRAS-B | 161 | ARG | HH12 | 1.9 |
| KRAS-A | 154 | ASP | OD2 | KRAS-B | 161 | ARG | HH22 | 1.9 |
| KRAS-A | 154 | ASP | OD2 | CRD-B | 175 | HIS | HE2 | 1.8 |
| KRAS-A | 161 | ARG | HH11 | KRAS-B | 154 | ASP | OD1 | 2.4 |
| KRAS-A | 161 | ARG | HH11 | KRAS-B | 154 | ASP | OD2 | 1.7 |
| KRAS-A | 161 | ARG | HH21 | KRAS-B | 154 | ASP | OD2 | 2.2 |
| KRAS-A | 164 | ARG | O | KRAS-B | 135 | ARG | HH11 | 1.8 |
| KRAS-A | 169 | LYS | O | KRAS-B | 128 | LYS | HZ1 | 1.7 |
| CRD-A | 175 | HIS | HN | KRAS-B | 150 | GLN | OE1 | 2.0 |
| CRD-A | 175 | HIS | HE2 | KRAS-B | 154 | ASP | OD2 | 1.8 |
| **Model 4** | | | | | | | | |
| **Chain** | **Residue Number** | **Residue Name** | **Atom Name** | **Chain** | **Residue Number** | **Residue Name** | **Atom Name** | **Distance^*^ (Å)** |
| KRAS-A | 47 | ASP | OD1 | KRAS-B | 143 | GLU | HN | 1.9 |
| KRAS-A | 49 | GLU | OE1 | KRAS-B | 128 | LYS | HZ1 | 1.6 |
| KRAS-A | 49 | GLU | OE1 | KRAS-B | 131 | GLN | HE22 | 1.8 |
| KRAS-A | 128 | LYS | HZ1 | KRAS-B | 169 | LYS | O | 1.7 |
| KRAS-A | 128 | LYS | HZ2 | KRAS-B | 2 | THR | OG1 | 1.9 |
| KRAS-A | 128 | LYS | HZ3 | KRAS-B | 49 | GLU | OE1 | 1.6 |
| KRAS-A | 131 | GLN | HE22 | KRAS-B | 49 | GLU | OE1 | 1.8 |
| KRAS-A | 135 | ARG | HH12 | KRAS-B | 164 | ARG | O | 1.8 |
| KRAS-A | 135 | ARG | HH12 | KRAS-B | 167 | LYS | O | 2.5 |
| KRAS-A | 143 | GLU | HN | KRAS-B | 47 | ASP | OD1 | 1.9 |
| KRAS-A | 143 | GLU | OE1 | CRD-B | 143 | ARG | HH11 | 1.9 |
| KRAS-A | 150 | GLN | OE1 | CRD-B | 175 | HIS | HN | 2.1 |
| KRAS-A | 154 | ASP | OD1 | KRAS-B | 161 | ARG | HH12 | 1.8 |
| KRAS-A | 154 | ASP | OD2 | KRAS-B | 161 | ARG | HH12 | 2.2 |
| KRAS-A | 154 | ASP | OD2 | KRAS-B | 161 | ARG | HH22 | 1.6 |
| KRAS-A | 154 | ASP | OD2 | CRD-B | 175 | HIS | HE2 | 1.7 |
| KRAS-A | 161 | ARG | HH11 | KRAS-B | 154 | ASP | OD1 | 1.7 |
| KRAS-A | 161 | ARG | HH21 | KRAS-B | 154 | ASP | OD2 | 1.7 |
| KRAS-A | 169 | LYS | O | KRAS-B | 128 | LYS | HZ2 | 1.8 |
| CRD-A | 161 | ASN | OD1 | KRAS-B | 148 | THR | HG1 | 2.5 |
| CRD-A | 175 | HIS | HN | KRAS-B | 150 | GLN | OE1 | 2.0 |
| CRD-A | 175 | HIS | HE2 | KRAS-B | 154 | ASP | OD2 | 1.8 |
| **Model 5** | | | | | | | | |
| **Chain** | **Residue Number** | **Residue Name** | **Atom Name** | **Chain** | **Residue Number** | **Residue Name** | **Atom Name** | **Distance^*^ (Å)** |
| KRAS-A | 47 | ASP | OD1 | KRAS-B | 143 | GLU | HN | 1.8 |
| KRAS-A | 47 | ASP | O | KRAS-B | 127 | THR | HG1 | 2.0 |
| KRAS-A | 49 | GLU | OE1 | KRAS-B | 128 | LYS | HZ1 | 1.6 |
| KRAS-A | 49 | GLU | OE1 | KRAS-B | 131 | GLN | HE22 | 1.8 |
| KRAS-A | 128 | LYS | HZ1 | KRAS-B | 169 | LYS | O | 1.7 |
| KRAS-A | 128 | LYS | HZ2 | KRAS-B | 2 | THR | OG1 | 1.9 |
| KRAS-A | 128 | LYS | HZ3 | KRAS-B | 49 | GLU | OE1 | 1.6 |
| KRAS-A | 131 | GLN | HE22 | KRAS-B | 49 | GLU | OE1 | 1.8 |
| KRAS-A | 135 | ARG | HH12 | KRAS-B | 164 | ARG | O | 2.2 |
| KRAS-A | 143 | GLU | HN | KRAS-B | 47 | ASP | OD1 | 2.2 |
| KRAS-A | 154 | ASP | OD1 | KRAS-B | 161 | ARG | HH12 | 1.7 |
| KRAS-A | 154 | ASP | OD2 | KRAS-B | 161 | ARG | HH22 | 1.7 |
| KRAS-A | 154 | ASP | OD2 | CRD-B | 175 | HIS | HE2 | 1.7 |
| KRAS-A | 161 | ARG | HH11 | KRAS-B | 154 | ASP | OD1 | 2.0 |
| KRAS-A | 161 | ARG | HH11 | KRAS-B | 154 | ASP | OD2 | 2.2 |
| KRAS-A | 161 | ARG | HH21 | KRAS-B | 154 | ASP | OD2 | 1.7 |
| KRAS-A | 164 | ARG | O | KRAS-B | 135 | ARG | HH11 | 2.0 |
| KRAS-A | 169 | LYS | O | KRAS-B | 128 | LYS | HZ2 | 1.8 |
| CRD-A | 143 | ARG | HH11 | KRAS-B | 143 | GLU | OE1 | 2.0 |
| CRD-A | 175 | HIS | HN | KRAS-B | 150 | GLN | OE1 | 2.3 |
| CRD-A | 175 | HIS | HE2 | KRAS-B | 154 | ASP | OD2 | 1.8 |
| **Model 6** | | | | | | | | |
| **Chain** | **Residue Number** | **Residue Name** | **Atom Name** | **Chain** | **Residue Number** | **Residue Name** | **Atom Name** | **Distance^*^ (Å)** |
| KRAS-A | 47 | ASP | OD1 | KRAS-B | 143 | GLU | HN | 2.0 |
| KRAS-A | 47 | ASP | O | KRAS-B | 127 | THR | HG1 | 1.9 |
| KRAS-A | 49 | GLU | OE1 | KRAS-B | 128 | LYS | HZ1 | 1.6 |
| KRAS-A | 49 | GLU | OE1 | KRAS-B | 131 | GLN | HE22 | 1.8 |
| KRAS-A | 127 | THR | HG1 | KRAS-B | 47 | ASP | O | 2.4 |
| KRAS-A | 128 | LYS | HZ1 | KRAS-B | 169 | LYS | O | 1.8 |
| KRAS-A | 128 | LYS | HZ2 | KRAS-B | 2 | THR | OG1 | 1.8 |
| KRAS-A | 128 | LYS | HZ3 | KRAS-B | 49 | GLU | OE1 | 1.6 |
| KRAS-A | 131 | GLN | HE22 | KRAS-B | 49 | GLU | OE1 | 1.7 |
| KRAS-A | 135 | ARG | HH12 | KRAS-B | 164 | ARG | O | 2.2 |
| KRAS-A | 143 | GLU | HN | KRAS-B | 47 | ASP | OD1 | 1.8 |
| KRAS-A | 143 | GLU | OE1 | CRD-B | 143 | ARG | HH11 | 2.3 |
| KRAS-A | 148 | THR | HG1 | CRD-B | 161 | ASN | OD1 | 2.3 |
| KRAS-A | 150 | GLN | OE1 | CRD-B | 175 | HIS | HN | 2.3 |
| KRAS-A | 154 | ASP | OD2 | KRAS-B | 161 | ARG | HH12 | 1.7 |
| KRAS-A | 154 | ASP | OD2 | KRAS-B | 161 | ARG | HH22 | 2.5 |
| KRAS-A | 154 | ASP | OD2 | CRD-B | 175 | HIS | HE2 | 1.9 |
| KRAS-A | 161 | ARG | HH11 | KRAS-B | 154 | ASP | OD1 | 2.0 |
| KRAS-A | 161 | ARG | HH11 | KRAS-B | 154 | ASP | OD2 | 2.0 |
| KRAS-A | 161 | ARG | HH21 | KRAS-B | 154 | ASP | OD2 | 1.8 |
| KRAS-A | 164 | ARG | O | KRAS-B | 135 | ARG | HH11 | 2.0 |
| KRAS-A | 169 | LYS | O | KRAS-B | 128 | LYS | HZ2 | 2.5 |
| KRAS-A | 169 | LYS | O | KRAS-B | 128 | LYS | HZ3 | 2.2 |
| CRD-A | 175 | HIS | HN | KRAS-B | 150 | GLN | OE1 | 2.1 |
| CRD-A | 175 | HIS | HE2 | KRAS-B | 154 | ASP | OD2 | 1.8 |
| **Model 7** | | | | | | | | |
| **Chain** | **Residue Number** | **Residue Name** | **Atom Name** | **Chain** | **Residue Number** | **Residue Name** | **Atom Name** | **Distance^*^ (Å)** |
| KRAS-A | 47 | ASP | OD1 | KRAS-B | 143 | GLU | HN | 1.7 |
| KRAS-A | 47 | ASP | O | KRAS-B | 127 | THR | HG1 | 2.4 |
| KRAS-A | 49 | GLU | OE1 | KRAS-B | 128 | LYS | HZ1 | 1.6 |
| KRAS-A | 49 | GLU | OE1 | KRAS-B | 131 | GLN | HE22 | 2.1 |
| KRAS-A | 128 | LYS | HZ1 | KRAS-B | 169 | LYS | O | 1.8 |
| KRAS-A | 128 | LYS | HZ2 | KRAS-B | 2 | THR | OG1 | 1.9 |
| KRAS-A | 128 | LYS | HZ3 | KRAS-B | 49 | GLU | OE1 | 1.6 |
| KRAS-A | 131 | GLN | HE22 | KRAS-B | 49 | GLU | OE1 | 1.8 |
| KRAS-A | 135 | ARG | HH12 | KRAS-B | 164 | ARG | O | 1.7 |
| KRAS-A | 143 | GLU | HN | KRAS-B | 47 | ASP | OD1 | 2.1 |
| KRAS-A | 150 | GLN | HE21 | CRD-B | 174 | GLU | OE1 | 1.7 |
| KRAS-A | 154 | ASP | OD1 | KRAS-B | 161 | ARG | HH12 | 1.7 |
| KRAS-A | 154 | ASP | OD2 | KRAS-B | 161 | ARG | HH22 | 1.8 |
| KRAS-A | 154 | ASP | OD2 | CRD-B | 175 | HIS | HE2 | 1.8 |
| KRAS-A | 161 | ARG | HH11 | KRAS-B | 154 | ASP | OD1 | 2.4 |
| KRAS-A | 161 | ARG | HH11 | KRAS-B | 154 | ASP | OD2 | 1.7 |
| KRAS-A | 164 | ARG | O | KRAS-B | 135 | ARG | HH11 | 2.1 |
| KRAS-A | 169 | LYS | O | KRAS-B | 128 | LYS | HZ2 | 1.9 |
| CRD-A | 175 | HIS | HN | KRAS-B | 150 | GLN | OE1 | 2.2 |
| CRD-A | 175 | HIS | HE2 | KRAS-B | 154 | ASP | OD2 | 1.8 |
| **Model 8** | | | | | | | | |
| **Chain** | **Residue Number** | **Residue Name** | **Atom Name** | **Chain** | **Residue Number** | **Residue Name** | **Atom Name** | **Distance^*^ (Å)** |
| KRAS-A | 2 | THR | OG1 | KRAS-B | 128 | LYS | HZ3 | 2.4 |
| KRAS-A | 47 | ASP | OD1 | KRAS-B | 143 | GLU | HN | 1.8 |
| KRAS-A | 47 | ASP | O | KRAS-B | 127 | THR | HG1 | 2.0 |
| KRAS-A | 49 | GLU | OE1 | KRAS-B | 128 | LYS | HZ1 | 1.5 |
| KRAS-A | 49 | GLU | OE1 | KRAS-B | 131 | GLN | HE22 | 1.8 |
| KRAS-A | 128 | LYS | HZ1 | KRAS-B | 49 | GLU | OE1 | 1.6 |
| KRAS-A | 128 | LYS | HZ2 | KRAS-B | 169 | LYS | O | 1.8 |
| KRAS-A | 128 | LYS | HZ3 | KRAS-B | 2 | THR | OG1 | 2.1 |
| KRAS-A | 131 | GLN | HE22 | KRAS-B | 49 | GLU | OE1 | 1.8 |
| KRAS-A | 135 | ARG | HH12 | KRAS-B | 164 | ARG | O | 1.9 |
| KRAS-A | 143 | GLU | HN | KRAS-B | 47 | ASP | OD1 | 1.8 |
| KRAS-A | 143 | GLU | OE1 | CRD-B | 143 | ARG | HH11 | 1.9 |
| KRAS-A | 150 | GLN | OE1 | CRD-B | 175 | HIS | HN | 2.0 |
| KRAS-A | 154 | ASP | OD1 | KRAS-B | 161 | ARG | HH12 | 2.4 |
| KRAS-A | 154 | ASP | OD2 | KRAS-B | 161 | ARG | HH12 | 1.8 |
| KRAS-A | 154 | ASP | OD2 | KRAS-B | 161 | ARG | HH22 | 2.2 |
| KRAS-A | 154 | ASP | OD2 | CRD-B | 175 | HIS | HE2 | 1.7 |
| KRAS-A | 161 | ARG | HH11 | KRAS-B | 154 | ASP | OD1 | 2.3 |
| KRAS-A | 161 | ARG | HH11 | KRAS-B | 154 | ASP | OD2 | 1.7 |
| KRAS-A | 161 | ARG | HH21 | KRAS-B | 154 | ASP | OD2 | 2.5 |
| KRAS-A | 164 | ARG | O | KRAS-B | 135 | ARG | HH11 | 2.2 |
| KRAS-A | 169 | LYS | O | KRAS-B | 128 | LYS | HZ2 | 1.8 |
| CRD-A | 175 | HIS | HE2 | KRAS-B | 154 | ASP | OD2 | 2.0 |
| **Model 9** | | | | | | | | |
| **Chain** | **Residue Number** | **Residue Name** | **Atom Name** | **Chain** | **Residue Number** | **Residue Name** | **Atom Name** | **Distance^*^ (Å)** |
| KRAS-A | 2 | THR | OG1 | KRAS-B | 128 | LYS | HZ3 | 2.0 |
| KRAS-A | 47 | ASP | OD1 | KRAS-B | 143 | GLU | HN | 1.7 |
| KRAS-A | 47 | ASP | O | KRAS-B | 127 | THR | HG1 | 2.3 |
| KRAS-A | 49 | GLU | OE1 | KRAS-B | 128 | LYS | HZ1 | 1.6 |
| KRAS-A | 49 | GLU | OE1 | KRAS-B | 131 | GLN | HE22 | 1.8 |
| KRAS-A | 128 | LYS | HZ1 | KRAS-B | 169 | LYS | O | 1.7 |
| KRAS-A | 128 | LYS | HZ2 | KRAS-B | 2 | THR | OG1 | 2.1 |
| KRAS-A | 128 | LYS | HZ3 | KRAS-B | 49 | GLU | OE1 | 1.6 |
| KRAS-A | 131 | GLN | HE22 | KRAS-B | 49 | GLU | OE1 | 1.8 |
| KRAS-A | 135 | ARG | HH12 | KRAS-B | 164 | ARG | O | 1.9 |
| KRAS-A | 143 | GLU | HN | KRAS-B | 47 | ASP | OD1 | 2.0 |
| KRAS-A | 150 | GLN | OE1 | CRD-B | 175 | HIS | HN | 2.4 |
| KRAS-A | 154 | ASP | OD1 | KRAS-B | 161 | ARG | HH12 | 1.7 |
| KRAS-A | 154 | ASP | OD2 | KRAS-B | 161 | ARG | HH12 | 2.3 |
| KRAS-A | 154 | ASP | OD2 | KRAS-B | 161 | ARG | HH22 | 1.8 |
| KRAS-A | 154 | ASP | OD2 | CRD-B | 375 | HIS | HE2 | 1.8 |
| KRAS-A | 161 | ARG | HH11 | KRAS-B | 154 | ASP | OD1 | 1.9 |
| KRAS-A | 161 | ARG | HH11 | KRAS-B | 154 | ASP | OD2 | 2.0 |
| KRAS-A | 161 | ARG | HH21 | KRAS-B | 154 | ASP | OD2 | 1.8 |
| KRAS-A | 164 | ARG | O | KRAS-B | 135 | ARG | HH11 | 1.9 |
| KRAS-A | 165 | LYS | HZ1 | KRAS-B | 162 | GLU | OE2 | 1.7 |
| KRAS-A | 169 | LYS | O | KRAS-B | 128 | LYS | HZ2 | 1.9 |
| CRD-A | 175 | HIS | HE2 | KRAS-B | 154 | ASP | OD2 | 1.8 |
| **Model 10** | | | | | | | | |
| **Chain** | **Residue Number** | **Residue Name** | **Atom Name** | **Chain** | **Residue Number** | **Residue Name** | **Atom Name** | **Distance^*^ (Å)** |
| KRAS-A | 2 | THR | OG1 | KRAS-B | 128 | LYS | HZ3 | 2.0 |
| KRAS-A | 47 | ASP | OD1 | KRAS-B | 143 | GLU | HN | 1.8 |
| KRAS-A | 47 | ASP | O | KRAS-B | 127 | THR | HG1 | 2.2 |
| KRAS-A | 49 | GLU | OE1 | KRAS-B | 128 | LYS | HZ1 | 1.6 |
| KRAS-A | 49 | GLU | OE1 | KRAS-B | 131 | GLN | HE22 | 1.9 |
| KRAS-A | 128 | LYS | HZ2 | KRAS-B | 169 | LYS | O | 2.0 |
| KRAS-A | 128 | LYS | HZ3 | KRAS-B | 49 | GLU | OE1 | 1.6 |
| KRAS-A | 131 | GLN | HE22 | KRAS-B | 49 | GLU | OE1 | 1.8 |
| KRAS-A | 135 | ARG | HH12 | KRAS-B | 164 | ARG | O | 2.1 |
| KRAS-A | 143 | GLU | HN | KRAS-B | 47 | ASP | OD1 | 2.0 |
| KRAS-A | 150 | GLN | HE21 | CRD-B | 174 | GLU | OE2 | 1.8 |
| KRAS-A | 154 | ASP | OD1 | KRAS-B | 161 | ARG | HH12 | 2.1 |
| KRAS-A | 154 | ASP | OD2 | KRAS-B | 161 | ARG | HH12 | 2.0 |
| KRAS-A | 154 | ASP | OD2 | KRAS-B | 161 | ARG | HH22 | 1.8 |
| KRAS-A | 154 | ASP | OD2 | CRD-B | 175 | HIS | HE2 | 1.8 |
| KRAS-A | 161 | ARG | HH11 | KRAS-B | 154 | ASP | OD1 | 2.0 |
| KRAS-A | 161 | ARG | HH11 | KRAS-B | 154 | ASP | OD2 | 2.0 |
| KRAS-A | 161 | ARG | HH21 | KRAS-B | 154 | ASP | OD2 | 1.9 |
| KRAS-A | 164 | ARG | O | KRAS-B | 135 | ARG | HH11 | 2.5 |
| KRAS-A | 169 | LYS | O | KRAS-B | 128 | LYS | HZ2 | 1.8 |
| CRD-A | 143 | ARG | HH11 | KRAS-B | 143 | GLU | OE1 | 1.8 |
| CRD-A | 175 | HIS | HN | KRAS-B | 150 | GLN | OE1 | 2.1 |
| CRD-A | 175 | HIS | HE2 | KRAS-B | 154 | ASP | OD2 | 1.9 |

**^*^** Cutoff distance to define a hydrogen bond is 2.5 Å.

**4. References**

[1] V. Tugarinov, P. M. Hwang, J. E. Ollerenshaw, L. E. Kay, *Journal of the American Chemical Society* **2003**, *125* (34), 10420, https://doi.org/10.1021/ja030153x.

[2] J. Iwahara, C. Tang, G. Marius Clore, *J Magn Reson* **2007**, *184* (2), 185, https://doi.org/10.1016/j.jmr.2006.10.003.

[3] F. Delaglio, S. Grzesiek, G. W. Vuister, G. Zhu, J. Pfeifer, A. Bax, *Journal of biomolecular NMR* **1995**, *6* (3), 277, https://doi.org/10.1007/bf00197809.

[4] B. A. Johnson, *Methods in molecular biology* **2004**, *278*, 313, https://doi.org/10.1385/1-59259-809-9:313.

[5] J. L. Battiste, G. Wagner, *Biochemistry* **2000**, *39* (18), 5355.

[6] G. C. P. van Zundert, J. Rodrigues, M. Trellet, C. Schmitz, P. L. Kastritis, E. Karaca, A. S. J. Melquiond, M. van Dijk, S. J. de Vries, A. Bonvin, *Journal of molecular biology* **2016**, *428* (4), 720, https://doi.org/10.1016/j.jmb.2015.09.014.

[7] J. Iwahara, C. D. Schwieters, G. M. Clore, *Journal of the American Chemical Society* **2004**, *126* (18), 5879, https://doi.org/10.1021/ja031580d.
